# Supplementary material for: The Daily Mile in practice: implementation and adaptation of the school running programme in a multiethnic city in the UK
Source: BMJ Open. 2021 Aug 2;11(8):e046655. doi: 10.1136/bmjopen-2020-046655 (PMC8330578; doi:10.1136/bmjopen-2020-046655)
Supplement: Supplementary data [file bmjopen-2020-046655supp001.pdf]

Daily Mile Survey

Welcome

The Daily Mile is being used in schools all over the UK. A team of researchers at the University of Leicester are doing some research on the Daily Mile in Leicester City.

The first step of this research is to find out how the Daily Mile is used in schools once they have signed up to the National Website.

This short survey is about the Daily Mile at your school.

Even if you do not use the Daily Mile at your school we would appreciate you answering some brief questions as to why it is not used in your school.

At the end, you can decide whether your school can be contacted by the research team from the University of Leicester regarding the second stage of the project.

Routen A, *et al*. *BMJ Open* 2021; 11:e046655. doi: 10.1136/bmjopen-2020-046655

Background

What is the name of your school?

Has your school signed up for the Daily Mile via the National Website?

- Yes
- No

For a typical week, on how many days per week is the Daily Mile run in your school?

- Never
- 1
- 2
- 3
- 4
- 5
- Other

Other (please comment):

Routen A, *et al. BMJ Open* 2021; 11:e046655. doi: 10.1136/bmjopen-2020-046655

Delivery

Why does your school not deliver the Daily Mile? Please select all that apply.

- ☐ Financial/funding reasons
- ☐ No support from teachers
- ☐ No support from parents
- ☐ Children already taking part in another active mile initiative
- ☐ Resource/staffing problems
- ☐ Difficulty in delivery
- ☐ Other

Difficulty in delivery (please comment):

Other (please comment):

Would your school benefit from some help or advice (from the Leicester City School Sport and Physical Activity Network) to deliver the Daily Mile?

- ○ Yes
- ○ No

Routen A, *et al. BMJ Open* 2021; 11:e046655. doi: 10.1136/bmjopen-2020-046655

Delivery

Does your school deliver the Daily Mile to all year groups?

- ○ Yes
- ○ No

Routen A, *et al. BMJ Open* 2021; 11:e046655. doi: 10.1136/bmjopen-2020-046655

Participation

Please select all applicable year groups that take part in the Daily Mile in your school.

- ☐ Foundation
- ☐ Year 1
- ☐ Year 2
- ☐ Year 3
- ☐ Year 4
- ☐ Year 5
- ☐ Year 6

Routen A, *et al. BMJ Open* 2021; 11:e046655. doi: 10.1136/bmjopen-2020-046655

Next Steps

If you would like to get involved in a research study by the University of Leicester about the Daily Mile please add your email address here or add the contact email of another staff member who can answer questions about the Daily Mile at your school.

Routen A, *et al. BMJ Open* 2021; 11:e046655. doi: 10.1136/bmjopen-2020-046655

Submit your answers

Please click 'Finish' to submit your answers.

Thank you!

Thank you for taking part in this short survey.

If you provided contact details we will be in touch soon regarding the next stage of the research.
