# Supplementary material for: The Daily Mile in practice: implementation and adaptation of the school running programme in a multiethnic city in the UK
Source: BMJ Open. 2021 Aug 2;11(8):e046655. doi: 10.1136/bmjopen-2020-046655 (PMC8330578; doi:10.1136/bmjopen-2020-046655)
Supplement: Supplementary data [file bmjopen-2020-046655supp002.pdf]

Example: Daily Mile Evaluation Coordinator/Head teacher Interview Guide

**Name of Teacher**:	**Date**:	**Start time**:
**School**:		**End Time**:

	Questions	To Probe	Checklist
**1**	**Reach**		
a)	How did you first hear about the Daily Mile?	Who are the key players, what steps had to be taken at the school to get buy-in.	
b)	How did you hear about the opportunity to sign up locally?		
c)	Why did you decide to sign your school up to the Daily Mile?		
d)	Describe the process of getting the school on board for this		
**2.**	**Level of Implementation**		
a)	Can you tell me how the Daily Mile is being run in your school?	Frequency and Duration	
**3.**	**Processes of Implementation [MISS OUT FOR NON-IMPLEMENTER & HEAD]**		
a)	How do the pupils move when they do the Daily Mile?	Walk, run, skip, hop, hold hands etc.	
b)	Where do you deliver the Daily Mile?	Playground, field, other site.	
b)	Please can you talk me through how you have approached each of the core DM principles in your school?	Show print out of DM principles. Adaptations e.g. skipping the DM, using non-curricular time	
c)	What are the practical preparations that you have done to deliver DM?	Clothing, weather, staffing, space.	
d)	How do you monitor pupil participation and pupil progress?<br>How do you use the Daily Mile in the teaching of any curriculum lessons?	Attendance, lap counting.	

Routen A, *et al. BMJ Open* 2021; 11:e046655. doi: 10.1136/bmjopen-2020-046655

	Questions	To Probe	Checklist
	Tell me about any adaptations you had to make to the school timetable in any way to deliver DM?		
e)	Beyond normal teaching time, tell me about any additional staff time or additional resources that have been used to deliver DM.		
4	**Implementation Drivers**		
a)	Tell me about any things which have made it difficult to deliver the Daily Mile in your school? (Barriers)	Pupil, teacher and school level issues. E.g. lack of staff to supervise, pupil disinterest, no SMT support.	
b)	Tell me about any things which have made it easier to deliver the Daily Mile in your school? (Facilitator)		
5	**Perceived Outcomes [MISS OUT FOR NON-IMPLEMENTER]**		
a)	Tell me about any changes in your pupils since doing the Daily Mile?	Positive changes such as improved attention in class, fitness etc.	
b)	Tell me about any changes in your teachers since doing the Daily Mile?	Negative changes such as misbehaviour in class.	
7	**Unintended Consequences**		
a)	Some schools have reported things changing in their schools that they were not expecting after starting TDM. Is there anything you can think of that changed?	We have examples from other schools. Let me tell you about them.<br>a. Other programmes have been stopped because TDM has started.<br>b. School decision makers are looking more favourably on physical activity within the school.<br>c. Time used for PE is being dedicated to TDM instead.	
b)	Think firstly for the pupils. Think then for the teachers. Think then for the school decision makers.		
8	**Maintenance [MISS OUT FOR NON-IMPLEMENTER]**		

	Questions	To Probe	Checklist
a)	Can you tell me how much or if the DM is now part of the school culture at your school?	Extent of routine practice, community involvement (parents), leadership support, school policy etc.	
b)	Can you describe what will/or is your school doing to continue the Daily Mile in future	School policy, funding, external support etc.	
**9**	**Decision to stop [NON-IMPLEMENTER ONLY]**		
a)	Why did you decide not to start the Daily Mile in your school?	Probe pupil, teacher and pupil barriers as above.	
b)	Why did you decide to stop delivering the Daily Mile in your school?	Probe pupil, teacher and pupil barriers as above.	
**10**	**Additional Questions**		
a)	How would you describe your role within the Daily Mile at your school (are they an intervention agent?)		
b)	Tell me what support you have looked for/been offered/received. Is there anything more you would need?		
c)	The Daily Mile has been called “simple”. What do you think about that statement?	Set-up and maintenance; if anything is thought to not be simple follow-up on that.	
d)	You said that XXXX was not simple/a challenge, how have you overcome that issue? Anything else?		
e)	The Daily Mile has been called “inexpensive”. What do you think about that statement?	Set-up and maintenance; if anything is thought to cost follow-up on that.	
f)	What do you think is ‘success’ in terms of delivering the Daily Mile in your school?		
Is there anything else we haven’t already mentioned or talked about which you think would be useful to know? Do you have any questions?			

Routen A, *et al. BMJ Open* 2021; 11:e046655. doi: 10.1136/bmjopen-2020-046655
