# Supplementary figures and images for: The Daily Mile in practice: implementation and adaptation of the school running programme in a multiethnic city in the UK
Source: BMJ Open. 2021 Aug 2;11(8):e046655. doi: 10.1136/bmjopen-2020-046655 (PMC8330578; doi:10.1136/bmjopen-2020-046655)

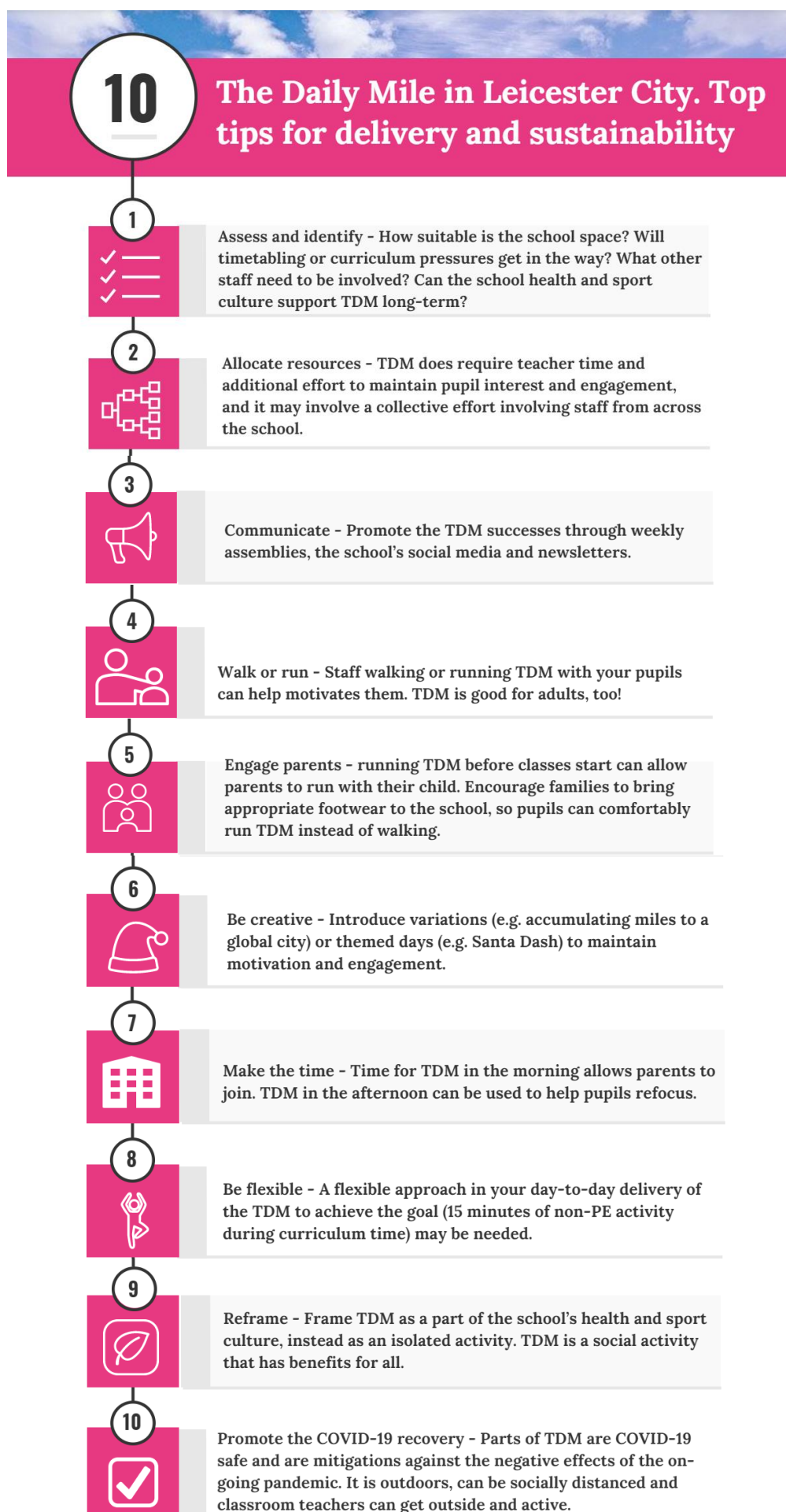

Supplement: Supplementary data [file bmjopen-2020-046655supp003.pdf]
