# Supplementary material for: The Daily Mile in practice: implementation and adaptation of the school running programme in a multiethnic city in the UK
Source: BMJ Open. 2021 Aug 2;11(8):e046655. doi: 10.1136/bmjopen-2020-046655 (PMC8330578; doi:10.1136/bmjopen-2020-046655)
Supplement: Author's manuscript [file bmjopen-2020-046655.draft_revisions.pdf]

BMJ Open

BMJ Open is committed to open peer review. As part of this commitment we make the peer review history of every article we publish publicly available.

When an article is published we post the peer reviewers' comments and the authors' responses online. We also post the versions of the paper that were used during peer review. These are the versions that the peer review comments apply to.

The versions of the paper that follow are the versions that were submitted during the peer review process. They are not the versions of record or the final published versions. They should not be cited or distributed as the published version of this manuscript.

BMJ Open is an open access journal and the full, final, typeset and author-corrected version of record of the manuscript is available on our site with no access controls, subscription charges or pay-per-view fees (http://bmjopen.bmj.com).

If you have any questions on BMJ Open's open peer review process please email info.bmjopen@bmj.com

BMJ Open

# “Things like The Daily Mile go on the backburner, don’t they?” Variable implementation of the schools running programme in a multi-ethnic city in the UK

| | |
|---|---|
| Journal: | *BMJ Open* |
| Manuscript ID | bmjopen-2020-046655 |
| Article Type: | Original research |
| Date Submitted by the Author: | 07-Nov-2020 |
| Complete List of Authors: | Routen, Ash; NIHR Applied Research Collaboration East Midlands<br>Gonzalez Aguado, Maria; University of Leicester<br>O' Connell, Sophie; University Hospitals of Leicester NHS Trust<br>Harrington, Deirdre; University of Leicester College of Life Sciences, Diabetes Research Centre |
| Keywords: | PUBLIC HEALTH, PREVENTIVE MEDICINE, PAEDIATRICS |
| | |

SCHOLARONE™
Manuscripts

*I, the Submitting Author has the right to grant and does grant on behalf of all authors of the Work (as defined in the below author licence), an exclusive licence and/or a non-exclusive licence for contributions from authors who are: i) UK Crown employees; ii) where BMJ has agreed a CC-BY licence shall apply, and/or iii) in accordance with the terms applicable for US Federal Government officers or employees acting as part of their official duties; on a worldwide, perpetual, irrevocable, royalty-free basis to BMJ Publishing Group Ltd ("BMJ") its licensees and where the relevant Journal is co-owned by BMJ to the co-owners of the Journal, to publish the Work in this journal and any other BMJ products and to exploit all rights, as set out in our licence.*

*The Submitting Author accepts and understands that any supply made under these terms is made by BMJ to the Submitting Author unless you are acting as an employee on behalf of your employer or a postgraduate student of an affiliated institution which is paying any applicable article publishing charge ("APC") for Open Access articles. Where the Submitting Author wishes to make the Work available on an Open Access basis (and intends to pay the relevant APC), the terms of reuse of such Open Access shall be governed by a Creative Commons licence – details of these licences and which Creative Commons licence will apply to this Work are set out in our licence referred to above.*

*Other than as permitted in any relevant BMJ Author's Self Archiving Policies, I confirm this Work has not been accepted for publication elsewhere, is not being considered for publication elsewhere and does not duplicate material already published. I confirm all authors consent to publication of this Work and authorise the granting of this licence.*

For peer review only

# "Things like The Daily Mile go on the backburner, don't they?" Variable implementation of the schools running programme in a multi-ethnic city in the UK

Ash Routen[1], María González Aguado[2], Sophie O' Connell[3], Deirdre M. Harrington[4,5*]

[1] NIHR Applied Research Collaboration East Midlands, Diabetes Research Centre, Leicester General Hospital, Gwendolen Road, Leicester, LE5 4PW.

[2] School of Communication, Media and Sociology, University of Leicester

[3] University Hospitals of Leicester NHS Trust, Leicester

[4] Diabetes Research Centre, University of Leicester

[5] School of Psychological Sciences and Health, University of Strathclyde

*Corresponding author email dh204@le.ac.uk

**Main Body Word Count:** 4896 (excluding tables)

For peer review only

## Abstract

**Objectives:** The aim of this study was to generate new evidence on how The Daily Mile (TDM), a popular school-based running programme in the UK, is implemented in a diverse and multi-ethnic city in the UK and also the barriers faced by non-implementer schools.

**Design:** Mixed method cross-sectional study (including survey data collection and qualitative interviews).

**Setting:** Primary schools in a multi-ethnic city in the East Midlands, UK.

**Participants:** 42 schools in Leicester city completed an online survey, and five teaching staff from five schools took part in follow-up semi-structured qualitative interviews.

**Results:** Overall, 37% of schools who completed the survey reported having never implemented TDM, and nearly all schools (96.6%) report delivering TDM on three or more days per week. Reported barriers included space limitations and safety issues, timetabling and curriculum pressures, and pupil and teacher attitudes. Facilitators of implementation were teacher engagement and school culture/ethos, communication of the initiative and delivery adaptations.

**Conclusions:** The findings from this study, based on data from schools in a multi-ethnic city in the UK, suggest that implementation of TDM is variable, and is influenced by a range of factors related to the school context, as well as the characteristics of TDM itself.

**Trial registration:** N/A


## Article Summary

### Strengths and limitations of the methods of this study

- Use of mixed methods in a multi-ethnic city.
- Views from non-implementer schools collected at two stages.
- Sample may represent schools who were particularly motivated to voice their opinion on implementation of TDM in their school.
- The focus on one city means that these findings cannot be generalised to all primary schools.


### Funding
This work was supported by a grant via the Wellcome Trust Institutional Strategic Support Fund at the University of Leicester. This work has been run by researchers from the University of Leicester and Leicester Diabetes Centre. It is supported by the NIHR Leicester Biomedical Research Centre and non-financial support has been provided by the *Cities Changing Diabetes* programme team in Leicester. *Cities Changing Diabetes Leicester* is a joint working project that is funded by and developed in collaboration by and between Leicester Diabetes Centre and Novo Nordisk. This work was also supported by the NIHR Applied Research Collaboration East Midlands (ARC EM).

### Competing interests
None to report




For peer review only - http://bmjopen.bmj.com/site/about/guidelines.xhtml

## Background

The Daily Mile (TDM) is a physical activity programme which challenges primary schools to have their pupils run a circuit of the playground, or similar, for a minimum of 15 minutes per day during class time. Since instigation in one Scottish primary school in 2012, TDM has 6,286 (as of November 2020) registered schools and nurseries in England. A recent data linkage study reported that one in five primary English schools have registered since 2012, and that registration is higher in urban areas, and for schools with higher levels of disadvantaged pupils (1).

Support for TDM has been such that its use was advocated in schools in the 2016 and 2018 UK Government childhood obesity strategy (2, 3) and in 2017 the Scottish Parliament wrote to all schools in Scotland to promote its uptake (4). Despite policy and funding support, evidence on the TDM effectiveness and context-specific operation is only emerging. A randomised controlled trial conducted in Birmingham, England, examined effectiveness of TDM on BMI z-score and undertook a subsequent economic analysis (5).

Regional qualitative and ethnographic studies have examined how schools implement TDM and what facilitates or constrains delivery from the perspectives of head teachers, teaching staff, pupils, parents and school governors as well as stakeholders including public health practitioners. These were conducted in Scotland (6, 7), Wales (8), South London (9) and rural England (10) and represent the views of schools from a variety of deprivation and geographic locations. The qualitative investigations of delivery and implementation (6-10) have largely focused on perceptions of teachers and schools who currently deliver TDM. There is a gap in knowledge from schools who have chosen not to implement TDM, or those who have tried with limited success.

The aim of this evaluation was to generate new evidence on how TDM is implemented in a ethnically and socio-economically diverse city and also the barriers faced by non-implementer schools. The objectives were to describe (a) TDM adoption and reach; (b) to uncover key barriers and facilitators to TDM implementation, and (c) to identify other key issues relating to how TDM was being implemented in Leicester, UK.

## Methods

### Study design

We undertook a mixed method two-stage evaluation during the 2018/19 and 2019/20 academic years. Stage 1 consisted of a city-wide cross-sectional online survey, while stage 2 was an interview with primary schools who completed the first survey. Ethics approval was obtained from the University of Leicester College of Life Sciences Concerning Human Subjects (non-NHS). A consent statement was included on the opening page of the online surveys. Individuals being interviewed provided written informed consent.

### Setting and population



For peer review only - http://bmjopen.bmj.com/site/about/guidelines.xhtml

Leicester (a multi-ethnic city in the Midlands, UK) is a microcosm of the UK due to its socioeconomic diversity and proximity to suburban and rural areas. TDM in primary schools is supported by Leicester City Council strategies and schools are encouraged to sign-up via the City Council intranet and though local primary teacher meetings.

**Patient and public involvement**

This work was informed by the Public Health team at Leicester City Council, and recruitment was supported by the Leicester City School Sport and Physical Activity Network (SSPAN). No other patient or public involvement took place.

**Stage 1: City-wide survey**

The TDM co-ordinators (or PE leads) of all city primary schools were emailed by SSPAN with the initial online survey link, and the survey was also advertised on local authority schools intranet and at teacher meetings. There were eight questions on TDM implementation and experiences, if any, of TDM. For example, respondents were asked for a typical week, on how many days per week TDM is run in their school and what year groups it is delivered to. Schools reporting no delivery of TDM were asked for reasons for non-delivery. Respondents could add free text comments, and they were asked whether they would be happy to be contacted for a follow-up interview.

Stage 2 recruited schools that had adopted and utilised TDM (i.e. implementer schools), and schools that initiated TDM but had failed to maintain implementation (i.e. non-implementers).

**Stage 2: Follow-up interview**

The contact person in ‘implementer’ schools was invited to a face-to-face interview. Interviews were conducted in schools by one trained researcher (AR) following a semi-structured topic guide. Interviews were recorded on a digital recorder and transcribed verbatim by an external transcription company. Transcripts were anonymised and identifiable information removed.

**Analysis**

A thematic analysis (11) was completed by one trained qualitative researcher (MGA); this was chosen to explore interviewees’ perceptions on the implementation of the TDM at their schools. An inductive approach to analysis was followed. Transcripts were read and re-read to identify relevant features. Interview data were systematically coded at the descriptive and semantic level using Nvivo (version 11.4.3).

**School data**

School sociodemographic characteristics (size, geographic location, ethnic mix) were taken from School Census records (January 2019). Deprivation data of the ward (locality division for electoral and planning purposes) in which the school is situated is based on Index of Multiple Deprivation (IMD) 2019.

## Results

As of winter 2019 there were 83 primary schools available to participate in TDM in Leicester. Of these, 42 (50.6%) completed the stage 1 survey (Table 1). Of these, 19 schools responded with willingness to take part in the interview (stage 2).



For peer review only - http://bmjopen.bmj.com/site/about/guidelines.xhtml

**Table 1.** Characteristics of the schools involved in Stage 1 (n = 42)

| | Implementers (n = 25) | Non-implementers/ other (n = 17) |
|---|---|---|
| **Pupil number (range)** | 401 (178 – 608) | 440 (108 – 747) |
| **% BME pupils (range)** | 49.8 (21.5 – 97.8) | 71.5 (20.2 – 98.0) |
| **% FSM (range)** | 23.4 (5.1 – 44.9) | 17.9 (108 – 758) |
| **% in Q1 (range)** | 40.0 (0 – 100) | 24.5 (0 – 61.1) |
| **% in IDACI Q1 (range)** | 48.9 (10.8 – 100) | 35.7 (9.2 – 69.6) |

Note: BME – black and minority ethnicity; FMS – free school meals; Q1 = quartile one (most deprived) of IMD; IDACI Q1 **-** Income Deprivation Affecting Children Index Quartile one (most deprived).

***Implementation level and type***

Of the schools that completed the survey, 40.5% (17/42) reported never running TDM. Of those who did implement it (59.5%; 25/42), 56.0% (14/25) reported running it five days/week, 4.0% (1/25) on four days, 36.0% (9/25) on three days, and 4.0% (1/25) on two days/week.

Table 2 presents the proportions of schools delivering TDM to various combinations of year groups. For example, 69% (20/29) of schools implementing TDM deliver it to all year groups from foundation year, to year 6. Schools reported in the survey that TDM delivery "*varies for different year groups*" and the "*amount actually varies greatly between classes and teachers.*"

**Table 2.** Proportions delivering to year groups

| **Year groups** | **%** |
|---|---|
| Foundation year to year 6 | 68.0 |
| Year 1 to year 6 | 4.0 |
| Foundation year only | 4.0 |
| Year 2 only | 8.0 |
| Year 4 only | 4.0 |
| Year 3 to year 6 | 12.0 |

***Stage 2 - Qualitative themes***

Interviews were conducted with TDM coordinators at five primary schools (see school characteristics in Table 3). Two of the five schools either did not deliver TDM or had previously delivered and discontinued after a few months. One of the schools delivered TDM before school at a local cricket ground (which they pay for) and parents sometimes watch the children.

**Table 3.** Characteristics of the schools involved in Stage 2



For peer review only - http://bmjopen.bmj.com/site/about/guidelines.xhtml

| School | School pupil number | % BAME | % FSM | Deprivation of ward | TDM Delivery | Contact person's role within school |
|---|---|---|---|---|---|---|
| 1 | 596 | 51.5 | 7.4 | Quintile 4 | No, "it was done sporadically for a few months" | Higher level teaching assistant and PE co-ordinator |
| 2 | 379 | 59.4 | 25.3 | Quintile 1 | Yes | Senior management team |
| 3 | 366 | 24.6 | 25.1 | Quintile 1 | Yes | PE co-ordinator and class teacher |
| 4 | 235 | 49.8 | 20.4 | Quintile 2 | No, The Daily Boost as an alternative is on-going | PE and history co-ordinator and class teacher |
| 5 | 468 | 40.6 | 23.4 | Quintile 2 | Yes although The Daily Boost is used with years 1 and 2 | PE Teacher |

Note: Deprivation of ward is based on IMD where a lower quintile is the most deprived; BAME = Black, Asian and Minority Ethnic group; FSM = free school meals.

Themes emerged through the aggrupation of representative subthemes that reflected the research questions, as well as new emergent features in the data. The iterative comparison of the responses in the interviews resulted in a coding framework consisting of five themes: 'Barriers to the delivery of TDM', 'facilitators of the TDM', 'tailoring physical activity offerings', 'perceived benefits' and 'measuring TDM success'. The description of these themes and the content of sub-themes are below.

## 1) Barriers to the delivery of TDM

### 1.1 Characteristics and functioning of the school

Three types of barriers related to the characteristics and functioning of the school were identified: space and safety concerns, pressures to deliver the National Curriculum and timetabling difficulties.

Four of the five interviewees affirmed that the lack of a safe and appropriately sized playground or field complicates the running of TDM, while three reported not having adequate space for the optimal running of TDM. Safety concerns relating to the quality of the space where pupils run also emerged as a possible implementation barrier. These concerns were related to the safety of the playground during adverse weather (for instance, puddles or icy pavement), that some pupils from disadvantaged families don't have appropriate footwear for all weather conditions and the risk of having a high number of pupils running at the same time in a small playground:

> *"(…) obviously, if we took a few classes down at a time health and safety becomes a little bit of an issue because we've got benches in the way, we've got bits around the playground so trying to get the boys not to be racing round and the girls."*



For peer review only - http://bmjopen.bmj.com/site/about/guidelines.xhtml

(School 1)

The description of space as an important barrier resonates with the free text qualitative data from non-implementer schools in Stage 1. These schools reported lack of space as one of the primary reasons for not delivering TDM. Some schools, especially those with larger pupil numbers, feel that they do not have enough space to deliver TDM: *"Due to the space and the size of the school. We only have a playground and 480 children. When PE is on there is a lack of space due to safety."*

A second barrier within this theme is the teachers/pupil timetables. Four schools found it difficult to fit TDM in their timetables without negatively impacting other teaching duties, such as assessments and lessons. In addition, delivering TDM on a daily basis makes one of the lessons fifteen minutes shorter.

> *"Well, in a week where you just think ahh, I've got so much to get through and we spent longer doing this so now we're running behind and it feels like that a lot of the time for a lot of us that we're always trying to fit more in than really we can. So, for some people. The Daily Mile is a thing too far, sadly."*

(School 2)

Stage 1 participants also highlighted the difficulties including TDM in the school's timetable as a barrier to delivery: "*Teachers timetables are so full with regular lessons and other initiatives that The Daily Mile would be too much of a burden to ask them to fit as well*" and "*They [pupils] already do a lot, don't know where it would go in the timetable*."

The third barrier within this theme was the pressure to deliver the National Curriculum. TDM coordinators intimated that delivering the curriculum is more important than pupils' daily physical activity, which could constrain TDM implementation. But it is important to note that two interviewees contend that TDM's impact on curriculum delivery is minimal, as having a physical activity break helps pupils focus. The excerpt below expresses interviewees' perception that the pressure to deliver the curriculum might be a barrier to the adoption and implementation of TDM:

> *"Even four- year olds are being baselined now [inaudible] timetables and all of this is like ways in which school will be held accountable by Ofsted [inaudible]. So, things like The Daily Mile go on the backburner, don't they? Which is a shame."*

(School 4)

### 1.2 Pupils' and teachers' attitudes

The second type of barrier related to pupils' and teachers' attitudes towards physical activity, the outdoors and TDM. Interviewees considered that teachers' personal attitudes towards physical activity and being outdoors influences the implementation of TDM:

> *"I think that is the biggest barrier* [school's space] *and then some people's lack of enthusiasm for being outside and being active which you get quite…if you have a range of staff that's what you're going to get"*

(School 2)



For peer review only - http://bmjopen.bmj.com/site/about/guidelines.xhtml

### 1.3 TDM in practice

The main sub-theme was that TDM is "not as simple" in practice. Although interviewees agreed that the TDM is easy to implement, three affirmed that it is not exempt of costs and implementation difficulties. They suggested TDM takes more than fifteen minutes, and involves additional costs for TDM to run autonomously. As outlined in TDM principles (12), the TDM has to be quick to deliver. However, the estimated fifteen minute delivery time does not include time needed to shepherd pupils to the playground, especially if the classroom lacks direct access to the open area. One teacher suggested the real implementation time can be up to thirty minutes.

Running TDM may involve additional costs. Here, interviewees were not referring to small expenses, such as footprints (painted on the playground to guide children) or tokens to count laps, but to staffing costs and teachers' time after work for additional preparation that optimal, engaging and long-term implementation requires. Interviewees explained that they invested time outside their working hours to collate pupils' times or create pupil progress certificates. One of the interviewees reported that TDM delivery required the collaboration and practical support of numerous teachers and other school staff:

> *"It does need managing so we have members of staff out. So, dinner ladies, lunch time supervisors and the class teachers now come out to manage because people can get into little groups and cause little bits of aggro so we try and nip that in the bud as much as possible."*
>
> (School 5)

## 2) Facilitators of TDM delivery

### 2.1 Schools' existing health and sport culture/ethos and teacher motivation

The approach of the school to health and physical activity can facilitate the long-term success of TDM in a school. All interviewees agreed that the TDM builds into, and contributes to, the wider culture or ethos of health and wellbeing within the school. Four interviewees described TDM as part of the school's health and sports culture, as an element of the "school's values":

> *"Yeah, part of our values is that actually being active and healthy is important and I think we do promote being healthy, our children get an opportunity to do cooking which they don't in a lot of schools, which is great, and we promote healthy eating through that but I think just being active, encouraging that would be, yeah, for it* [TDM] *to be part of our school values would be good if it was a greater part of our school values."*
>
> (School 2)

Interviewees mentioned several times the importance of the support from the school in running TDM. By support from the school, interviewees referred to the schools' interest in introducing TDM and the acknowledgment that pupils' daily physical activity and the TDM are an important part of the school's values and activities. Teachers' motivation and the development of a health and sports culture within the school can positively influence TDM delivery. Interviews responses showed that teachers' involvement in the delivery of TDM impacts pupils' performance. Teachers running alongside and cheering pupils was reported to be a motivator and aid for pupil engagement.



For peer review only - http://bmjopen.bmj.com/site/about/guidelines.xhtml

### 2.2 Communication of TDM

Interviews revealed that communicating the TDM is a key to facilitating implementation. Schools make use of their communication channels (e.g. newsletters), to communicate that they are running TDM. For some schools, the school's weekly assembly is a key communication space to engage students with TDM and reinforce the role of TDM as part of the school's health culture. During the school assembly, for instance, weekly results of TDM competition (see 2.4 below) are communicated and progress certificates handed out:

> *"(…) if they're doing the right thing then they'll keep going and some children do eight laps, some children do two and at the end of the week I record it all, put it on a spreadsheet so I've got the data of who is doing what, when. We look at celebrating that in assembly with the class that goes the furthest."*
>
> (School 5)

### 2.3 Ability to modify their limited space

Interviewees explained that small modifications in playgrounds to mark the path or giving pupils a token to count laps can facilitate TDM. One interviewee reported that her school have painted footprints on the playground to make running TDM more intuitive. Interviewees regard the use of tokens and clickers to count laps as positive:

> *"Yes, something I hadn't thought of but think could be quite useful, sometimes if children could have clickers, you know when they count people into things, so if they could click every time they do a lap that might be good in terms of resources."*
>
> (School 2)

### 2.4 "Keep it fresh"

As noted above, if schools deliver TDM according to the core principle of simplicity (getting pupils to run or walk for fifteen minutes per day) pupils "*would lose interest in it after a week quite quickly*." All the interviewees explained that they had to introduce some variations or adapt TDM to maintain pupils' engagement with the initiative in the long-term. Coordinators developed diverse strategies to maintain pupils' motivation and interest in TDM. Strategies included introducing an element of competition (inter class competitions), monitoring and rewarding pupils' progress and "*keeping the TDM fresh*". As one interviewee explains, they made TDM complicated to engage pupils:

> *"I've made it complicated to suit what I want to get out of it. If I want my children to do something I have to have that element of competition (…) So yeah, I'd agree with that and I just make a rod for my own back to make it more enjoyable for the children, I guess. For my children because a sleepy village in Scotland has a different cohort to inner city Leicester."*
>
> (School 5)

Monitoring pupils' times and distance ran was regarded as motivating for pupils by coordinators. It is necessary to clarify that that monitoring does not aim at promoting individual competition or creating an 'elite' group of fitter pupils, but to encouraging self-improvement by presenting TDM as a collective endeavour. Regarding competitivity among pupils it is important to note that the school 1 interviewee contended that competition does not always work, as many pupils are not competitive.



For peer review only - http://bmjopen.bmj.com/site/about/guidelines.xhtml

For peer review only

To ensure pupils' interest and engagement with TDM in the long-term, interviewee number 5 explained that teachers and schools have to 'keep TDM fresh' to motivate and engage pupils. 'Keep TDM fresh', in this case, means updating the initiative on an annual basis, so pupils maintain their interest and motivation year after year. "*Keeping TDM fresh*" requires modifying the initiative at two levels: 1) Introducing changes in the TDM implementation design for the academic year (i.e. measuring the accumulative time and translating into distance on a map, i.e. "*this year [we] have run the distance between Spain and the UK*"; introducing a system of houses, pupils run for their house to win etc.) and 2) measures to implement TDM on a daily basis (i.e. monitoring pupils time, use of tokens, mark the distance pupils have to run).

**3) Tailoring their physical activity offering**

Schools are interested in delivering physical activity programmes to keep their pupils active daily. Schools adapted programmes to overcome internal barriers and engage pupils. Four of the five interviewed schools combine different daily exercise programmes overcoming spatial, timetable and curriculum barriers.

Two interviewees introduced two alternative physical activities to deliver instead of TDM in adverse weather in the absence of a covered playground. Another school used different physical activity initiatives in different school years according to pupils' age to create a culture of health and sports in the school. In this school, younger pupils do The Daily Boost - an initiative encouraging fifteen minutes of organised physical activity at the school, this can be running, walking, cycling, dancing or any physical activity pupils may like - while TDM is usually implemented among the older pupils, so younger pupils associate transitioning to higher years with running TDM:

> *"Oh yeah, so we rolled out The Daily Boost to kind of…so The Daily Mile was running quite happily and then we've gone well, we need to do something for the younger children to get them. So, it's not just a shock at Year 3, so it slowly integrates into their school life. So yeah, we have started to roll that out and that was rolled out at the start of this year."*
>
> (Interview, 5)

Schools, hence, create their "*own version*" of the TDM to successfully implement the TDM according to the necessities and characteristics of their schools and pupils:

> *"Yes, so we've created our own version to keep the children engaged. You see with the skipping, there's so many different variations. They can learn tricks, they can do spins. Like we've got a few big ropes so they're doing different games, different activities with the big ropes, they just love having little competitions against each other to see who can skip for the longest, it's just a little bit more, they can vary it so much whereas with the Daily Mile you can't really, there's not that much to vary apart from saying to them let's see if you can increase your time but again."*
>
> (School 1)

The interview findings reflect the Stage 1 survey reporting that schools, despite the mentioned barriers, try to develop strategies to help their situation. For instance, by applying for funds to improve their space as in the excerpt *"Grant applied for to fund a track"* or to ensure their pupils could still be active in a similar fifteen minute window using skipping ropes. Schools, in particular, were implementing The Daily Boost which is seen as more practical and offered flexibility for their size and space.


For peer review only - http://bmjopen.bmj.com/site/about/guidelines.xhtml

## 4) Perceived benefits of TDM

All interviewees reported that TDM benefits their pupils. The benefits can be classified into physical and behavioural. For physical it is through the improvement in fitness or by encouraging them to practice sports outside the school. Two teachers reported that TDM improved the physical condition of their pupils and increased their physical activity levels.

Interviewees 1 and 5 explained that TDM acted as a catalyser of pupils like for being physically active and sports and making healthier choices. Interviewee 5 tells that one pupil started to become interested in practising sports after school as a consequence of the implementation of TDM at the school:

> *"So, I've got one family who she started doing cross country in Year 3, she's won the medal, so now she goes running with her mum and I didn't expect that to happen and now she loves it, she loves running and she's been successful at it and it's kind of given her an in to other sports. Now she's part of the girls football team, she plays cricket, she gets stuck in with everything and the running was the catalyst."*
>
> (School 5)

The interviewees agreed in that TDM not only have physical benefits for pupils, it also brings about behavioural and social benefits. Four interviewees agreed in that that running TDM improved pupils' attention and concentration. As they explained, having a break for physical exercise help them re-focus on their learning. Interviewee 1 also affirmed that TDM help pupils to manage their anger:

> "(…) we have a few children that perhaps have behavioural problems, anger problems and you know, they go out and it does help them. They'll say to you that it helps them and it helps them focus a little bit more if they have that break, go out, do that, come back in and they feel that…"
>
> (School 1)

## 5) Measuring success

Although interviewees reported the TDM to be beneficial, there is no evidence of the benefits of the TDM beyond teachers' own perceptions. The interviewees' focused on "keeping pupils active" putting the ways in which pupils do TDM in a secondary place. The interviewees described TDM as beneficial without giving excessive importance to the fact that some pupils (especially girls) walk instead of running or if they run in smaller laps if the playground is too busy. Interviewees described TDM as a successful initiative because it helps them to ensure that pupils are physically active ("The whole thing is keeping them active"):

> *"The most important thing for me is that it's done. If we're really squeezed for time and to be honest, sometimes we are and I'll say right, we're going to do half a mile."*
>
> (School 2)



For peer review only - http://bmjopen.bmj.com/site/about/guidelines.xhtml

## Discussion

### Main findings

Over a third of schools (37%) who completed the survey reported never implementing TDM, which equates to an adoption rate of 34% (29/83 schools). In relation to dose delivered, we over half (55.2%) of schools implemented TDM every day - a TDM recommendation at time of data collection. Subsequently TDM recommend delivery on at least three days, which nearly all schools (96.6%) met. The majority (69%) report delivering TDM to all year groups from foundation year, to Year 6, suggesting there is good reach across the school. However teachers acknowledged delivery varies within and across year groups.

Our results from an ethnically and socioeconomically diverse city make a useful addition to the extant literature. Although it was highlighted that what works in a rural area in Scotland would not work for a multi-ethnic city like Leicester no themes related to ethnicity emerged in our study. However, Stage 1 data show the percentage of schools in the lowest deprivation quartile was higher in the implementer than non-implementer schools which supplements data showing that TDM-registered schools had more disadvantaged pupils (1).

TDM coordinators conveyed a range of class, teacher and organisational level issues they felt constrained the delivery of TDM including space limitations and safety concerns, national curriculum pressures, and timetabling difficulties. In relation to pupils and teachers, attitudes toward physical activity and the outdoors appeared to act as a constraint to implementation. Facilitators of delivery included coordinating teachers' motivation, and the existing school ethos and culture regarding physical activity and wellbeing. Finally, the need to adapt the delivery of TDM to maintain pupil motivation was identified as an important issue, as was the creation of alternative physical activity programmes, to supplement TDM, or to deliver in place of TDM, due to the needs of the given school context.

### Strengths and limitations of the study

Strengths of this study include the use of mixed methods, with the survey providing information on the implementation of TDM across a wide-range of schools in a multi-ethnic city. Only one extant study has examined TDM implementation at a city/borough level (9). This enabled identification of variation in practice, including schools who did not implement TDM, which was further examined by following-up a small number of schools for more detailed understanding via interviews. A limitation of this approach however is that our interview sample may represent schools who were particularly motivated (schools self-selected availability for inclusion in further research in the survey) to voice their opinion on implementation of TDM in their school i.e. successful implementer schools, or conversely those with difficulties or no implementation. In addition, as is inherent within qualitative work, and the focus on a densely populated urban area, these findings cannot be generalised to all primary schools.

### Comparison to existing literature

The adoption rate of 34% is lower than the 48% reported from a TDM evaluation in London schools (9). Two previous studies have detailed the dose of TDM delivered in schools. Focused on one primary school in the East Midlands, Harris et al. (10) reported key stage teachers delivered TDM on 86% of days, with 95% of pupils receiving the intervention on those days. Hanckel et al. (9) qualitatively summarised dose information, suggesting TDM was not delivered every day in most schools/classes, and that it was viewed as interchangeable with other physical health interventions within the school. To our knowledge there is no other existing data on reach of TDM delivery across year groups.

The barriers of space limitations, timetabling and pressures to deliver the curriculum are

consistent with findings from previous TDM evaluations (7-9) as well as being common barriers to the delivery of other physical activity initiatives or policies in schools (13, 14). Likewise teacher buy-in (i.e. whether they thought it worthwhile and whether they liked it or not) has been reported as a barrier in other school-based running programmes (15). In relation to implementation facilitators, previous work on TDM has identified that teacher's active participation in TDM is important for enabling pupil engagement with the initiative (8) The importance of TDM aligning with the existing school culture and ethos towards physical activity and wellbeing, and therefore acting as a facilitator to implementation, has been highlighted in research on a similar school-based running programme in the UK (15).

## Implications for practice

When promoting TDM to diverse schools within a local authority area strategies to help schools adapt TDM or even create alternatives to keep pupils active for fifteen minutes according to the needs and preferences of their school are needed. We didn't find a need to adapt based on ethnic make-up of the school. One unique point related to disadvantage emerged whereby pupils shoes available were not suitable for all weathers. It is important that schools and stakeholders consider factors that support long-term implementation prior to starting TDM in a school. In particular consideration should be given to the existing implementation context within the school, such as the physical environment (space, playground options) organisational issues (timetabling, curriculum focus/pressure) staffing (teacher attitudes and motivation), as well as the schools culture and ethos regarding physical activity and health. These considerations are also relevant to any physical activity programme that is embedded in schools. It may also be important to emphasise to schools that in spite of apparent simplicity, TDM does require teacher time and additional effort to maintain pupil interest and engagement, and it may involve a collective effort involving staff from across the school until it can run autonomously. Finally, as is the case with Leicester city schools, it is clear there are ways to adapt TDM to facilitate delivery and allow children to participate in a vital fifteen minutes of physical activity that it distinct from PE and embedded in curriculum time.

## Conclusions and future research

The findings from this study, based on data from schools in a multi-ethnic city in the UK, suggest that implementation of TDM is variable, and is influenced by a range of factors related to the school context, as well as the characteristics of TDM itself. Future research should focus on understanding how to support schools in readiness for implementation of TDM and school-based running programmes, and learning from schools that implement adapted versions of the TDM, or alternative programmes with similar core principles.

## References

1. Venkatraman T, Honeyford K, Costelloe CE, Bina R, M F van Sluijs E, Viner RM, et al. Sociodemographic profiles, educational attainment and physical activity associated with The Daily Mile™ registration in primary schools in England: a national cross-sectional linkage study. Journal of Epidemiology and Community Health. 2020:jech-2020-214203.
2. UK Government. Childhood Obesity Strategy: A Plan for Action. 2016.
3. UK Government. Childhood Obesity Strategy: A Plan for Action, Chapter 2. 2018.
4. Scottish Government. Scotland: a Daily Mile Nation. 2017.
5. Breheny K, Passmore S, Adab P, Martin J, Hemming K, Lancashire ER, et al. Effectiveness and cost-effectiveness of The Daily Mile on childhood weight outcomes and wellbeing: a cluster randomised controlled trial. International Journal of Obesity. 2020:1-11.



For peer review only - http://bmjopen.bmj.com/site/about/guidelines.xhtml

6. Ryde GC, Booth JN, Brooks NE, Chesham RA, Moran CN, Gorely T. The Daily Mile: What factors are associated with its implementation success? PloS one. 2018;13(10).
7. Malden S, Doi L. The Daily Mile: teachers' perspectives of the barriers and facilitators to the delivery of a school-based physical activity intervention. BMJ open. 2019;9(3):e027169.
8. Marchant E, Todd C, Stratton G, Brophy S. The Daily Mile: Whole-school recommendations for implementation and sustainability. A mixed-methods study. PloS one. 2020;15(2):e0228149.
9. Hanckel B, Ruta D, Scott G, Peacock JL, Green J. The Daily Mile as a public health intervention: a rapid ethnographic assessment of uptake and implementation in South London, UK. BMC public health. 2019;19(1):1167.
10. Harris J, Milnes LJ, Mountain G. How 'The Daily Mile™'works in practice: A process evaluation in a UK primary school. Journal of child health care. 2019:1367493519880049.
11. Braun V, Clarke V. Using thematic analysis in psychology. Qualitative research in psychology. 2006;3(2):77-101.
12. The Daily Mile Foundation. 10 core principles. Your steps to Daily Mile success 2020 [Available from: https://thedailymile.co.uk/wp-content/uploads/2020/07/A4-Core-Principles.pdf.
13. Nathan N, Elton B, Babic M, McCarthy N, Sutherland R, Presseau J, et al. Barriers and facilitators to the implementation of physical activity policies in schools: a systematic review. Prev Med. 2018;107:45-53.
14. Routen AC, Johnston JP, Glazebrook C, Sherar LB. Teacher perceptions on the delivery and implementation of movement integration strategies: the CLASS PAL (physically active learning) Programme. International Journal of Educational Research. 2018;88:48-59.
15. Chalkley AE, Routen AC, Harris JP, Cale LA, Gorely T, Sherar LB. A retrospective qualitative evaluation of barriers and facilitators to the implementation of a school-based running programme. BMC Public Health. 2018;18(1):1189.

## Acknowledgements

We thanks Daxa Ralhan from Leicester City Council and Sarah Lansdowne from Leicester City School Sport and Physical Activity Network for assisting with school recruitment. We thank Andrew Fry and Anna Harding from the University of Leicester for administering the funding.

## Author contribution statement

AR, DH and SC conceived the original study design and methods. AR collected data and MGA led the qualitative analysis. MGA and DH discussed and refined the emerging codes and final themes. AR, DH and MGA wrote the first draft of the manuscript. All authors reviewed the manuscript and provided feedback and comments. All authors read and approved the final manuscript.

## Data sharing statement

Data sharing is not possible as it was not included in the consent forms. However, all reasonable requests for access the data for reanalysis will be considered. Please contact the corresponding author.

## Word Count

For peer review only

# Reporting checklist for cross sectional study.

Based on the STROBE cross sectional guidelines.

## Instructions to authors

Complete this checklist by entering the page numbers from your manuscript where readers will find each of the items listed below.

Your article may not currently address all the items on the checklist. Please modify your text to include the missing information. If you are certain that an item does not apply, please write "n/a" and provide a short explanation.

Upload your completed checklist as an extra file when you submit to a journal.

In your methods section, say that you used the STROBE cross sectionalreporting guidelines, and cite them as:

von Elm E, Altman DG, Egger M, Pocock SJ, Gotzsche PC, Vandenbroucke JP. The Strengthening the Reporting of Observational Studies in Epidemiology (STROBE) Statement: guidelines for reporting observational studies.

| | | Reporting Item | Page Number |
|---|---|---|---|
| **Title and abstract** | | | |
| Title | #1a | Indicate the study's design with a commonly used term in the title or the abstract | 2 |

For peer review only

| | | | |
|---|---|---|---|
| Abstract | #1b | Provide in the abstract an informative and balanced summary of what was done and what was found | 2 |
| **Introduction** | | | |
| Background / rationale | #2 | Explain the scientific background and rationale for the investigation being reported | 3 |
| Objectives | #3 | State specific objectives, including any prespecified hypotheses | 3 |
| **Methods** | | | |
| Study design | #4 | Present key elements of study design early in the paper | 3 |
| Setting | #5 | Describe the setting, locations, and relevant dates, including periods of recruitment, exposure, follow-up, and data collection | 3 |
| Eligibility criteria | #6a | Give the eligibility criteria, and the sources and methods of selection of participants. | 4 |
| | #7 | Clearly define all outcomes, exposures, predictors, potential confounders, and effect modifiers. Give diagnostic criteria, if applicable | N/A |
| Data sources / measurement | #8 | For each variable of interest give sources of data and details of methods of assessment (measurement). Describe comparability of assessment methods if there is more than one group. Give information separately for for exposed and unexposed groups if applicable. | 4 |

For peer review only

| | | | |
|---|---|---|---|
| Bias | #9 | Describe any efforts to address potential sources of bias | N/A |
| Study size | #10 | Explain how the study size was arrived at | 4 |
| Quantitative variables | #11 | Explain how quantitative variables were handled in the analyses. If applicable, describe which groupings were chosen, and why | 4 |
| Statistical methods | #12a | Describe all statistical methods, including those used to control for confounding | N/A |
| Statistical methods | #12b | Describe any methods used to examine subgroups and interactions | N/A |
| Statistical methods | #12c | Explain how missing data were addressed | N/A |
| Statistical methods | #12d | If applicable, describe analytical methods taking account of sampling strategy | N/A |
| Statistical methods | #12e | Describe any sensitivity analyses | N/A |

## Results

| | | | |
|---|---|---|---|
| Participants | #13a | Report numbers of individuals at each stage of study—eg numbers potentially eligible, examined for eligibility, confirmed eligible, included in the study, completing follow-up, and analysed. Give information separately for for exposed and unexposed groups if applicable. | 5 |
| Participants | #13b | Give reasons for non-participation at each stage | 5 |

For peer review only

| | | | |
|---|---|---|---|
| Participants | #13c | Consider use of a flow diagram | N/A |
| Descriptive data | #14a | Give characteristics of study participants (eg demographic, clinical, social) and information on exposures and potential confounders. Give information separately for exposed and unexposed groups if applicable. | Table 1, 3 |
| Descriptive data | #14b | Indicate number of participants with missing data for each variable of interest | N/A |
| Outcome data | #15 | Report numbers of outcome events or summary measures. Give information separately for exposed and unexposed groups if applicable. | N/A |
| Main results | #16a | Give unadjusted estimates and, if applicable, confounder-adjusted estimates and their precision (eg, 95% confidence interval). Make clear which confounders were adjusted for and why they were included | N/A |
| Main results | #16b | Report category boundaries when continuous variables were categorized | N/A |
| Main results | #16c | If relevant, consider translating estimates of relative risk into absolute risk for a meaningful time period | N/A |
| Other analyses | #17 | Report other analyses done—e.g., analyses of subgroups and interactions, and sensitivity analyses | N/A |
| **Discussion** | | | |
| Key results | #18 | Summarise key results with reference to study objectives | 12 |

For peer review only

| | | | |
|---|---|---|---|
| Limitations | #19 | Discuss limitations of the study, taking into account sources of potential bias or imprecision. Discuss both direction and magnitude of any potential bias. | 12 |
| | #20 | Give a cautious overall interpretation considering objectives, limitations, multiplicity of analyses, results from similar studies, and other relevant evidence. | 12 |
| Generalisability | #21 | Discuss the generalisability (external validity) of the study results | 13 |
| **Other Information** | | | |
| Funding | #22 | Give the source of funding and the role of the funders for the present study and, if applicable, for the original study on which the present article is based | 1 |

None The STROBE checklist is distributed under the terms of the Creative Commons Attribution License CC-BY. This checklist can be completed online using https://www.goodreports.org/, a tool made by the EQUATOR Network in collaboration with Penelope.ai

For peer review only

## Standards for Reporting Qualitative Research (SRQR)*

http://www.equator-network.org/reporting-guidelines/srqr/

### Title and abstract

| | Page/line no(s). |
|---|---|
| **Title** - Concise description of the nature and topic of the study Identifying the study as qualitative or indicating the approach (e.g., ethnography, grounded theory) or data collection methods (e.g., interview, focus group) is recommended | N/A |
| **Abstract** - Summary of key elements of the study using the abstract format of the intended publication; typically includes background, purpose, methods, results, and conclusions | 2 |

### Introduction

For peer review only

| | |
|---|---|
| **Problem formulation** - Description and significance of the problem/phenomenon studied; review of relevant theory and empirical work; problem statement | 3 |
| **Purpose or research question** - Purpose of the study and specific objectives or questions | 3 |

**Methods**

| | |
|---|---|
| **Qualitative approach and research paradigm** - Qualitative approach (e.g., ethnography, grounded theory, case study, phenomenology, narrative research) and guiding theory if appropriate; identifying the research paradigm (e.g., postpositivist, constructivist/ interpretivist) is also recommended; rationale** | 4 |
| **Researcher characteristics and reflexivity** - Researchers' characteristics that may influence the research, including personal attributes, qualifications/experience, relationship with participants, assumptions, and/or presuppositions; potential or actual interaction between researchers' characteristics and the research questions, approach, methods, results, and/or transferability | No |
| **Context** - Setting/site and salient contextual factors; rationale** | 3 |
| **Sampling strategy** - How and why research participants, documents, or events were selected; criteria for deciding when no further sampling was necessary (e.g., sampling saturation); rationale** | 3/4 |
| **Ethical issues pertaining to human subjects** - Documentation of approval by an appropriate ethics review board and participant consent, or explanation for lack thereof; other confidentiality and data security issues | 3 |
| **Data collection methods** - Types of data collected; details of data collection procedures including (as appropriate) start and stop dates of data collection and analysis, iterative process, triangulation of sources/methods, and modification of procedures in response to evolving study findings; rationale** | 3,4 |
| **Data collection instruments and technologies** - Description of instruments (e.g., interview guides, questionnaires) and devices (e.g., audio recorders) used for data collection; if/how the instrument(s) changed over the course of the study | 4 |
| **Units of study** - Number and relevant characteristics of participants, documents, or events included in the study; level of participation (could be reported in results) | 4 |
| **Data processing** - Methods for processing data prior to and during analysis, including transcription, data entry, data management and security, | 4 |

For peer review only

| | |
|---|---|
| verification of data integrity, data coding, and anonymization/de-identification of excerpts | |
| **Data analysis** - Process by which inferences, themes, etc., were identified and developed, including the researchers involved in data analysis; usually references a specific paradigm or approach; rationale** | 4 |
| **Techniques to enhance trustworthiness** - Techniques to enhance trustworthiness and credibility of data analysis (e.g., member checking, audit trail, triangulation); rationale** | |

**Results/findings**

| | |
|---|---|
| **Synthesis and interpretation** - Main findings (e.g., interpretations, inferences, and themes); might include development of a theory or model, or integration with prior research or theory | 6 onwards |
| **Links to empirical data** - Evidence (e.g., quotes, field notes, text excerpts, photographs) to substantiate analytic findings | No |

**Discussion**

| | |
|---|---|
| **Integration with prior work, implications, transferability, and contribution(s) to the field -** Short summary of main findings; explanation of how findings and conclusions connect to, support, elaborate on, or challenge conclusions of earlier scholarship; discussion of scope of application/generalizability; identification of unique contribution(s) to scholarship in a discipline or field | 12,13 |
| **Limitations** - Trustworthiness and limitations of findings | 12 |

**Other**

| | |
|---|---|
| **Conflicts of interest** - Potential sources of influence or perceived influence on study conduct and conclusions; how these were managed | 1 |
| **Funding** - Sources of funding and other support; role of funders in data collection, interpretation, and reporting | 1 |

*The authors created the SRQR by searching the literature to identify guidelines, reporting standards, and critical appraisal criteria for qualitative research; reviewing the reference lists of retrieved sources; and contacting experts to gain feedback. The SRQR aims to improve the transparency of all aspects of qualitative research by providing clear standards for reporting qualitative research.

For peer review only

**The rationale should briefly discuss the justification for choosing that theory, approach, method, or technique rather than other options available, the assumptions and limitations implicit in those choices, and how those choices influence study conclusions and transferability. As appropriate, the rationale for several items might be discussed together.

**Reference:**

O'Brien BC, Harris IB, Beckman TJ, Reed DA, Cook DA. **Standards for reporting qualitative research: a synthesis of recommendations.** *Academic Medicine*, Vol. 89, No. 9 / Sept 2014

DOI: 10.1097/ACM.0000000000000388

For peer review only

BMJ Open

# The Daily Mile in practice: Implementation and adaptation of the school running programme in a multi-ethnic city in the UK

| | |
|---|---|
| Journal: | *BMJ Open* |
| Manuscript ID | bmjopen-2020-046655.R1 |
| Article Type: | Original research |
| Date Submitted by the Author: | 28-Mar-2021 |
| Complete List of Authors: | Routen, Ash; NIHR Applied Research Collaboration East Midlands<br>Gonzalez Aguado, Maria; University of Leicester<br>O' Connell, Sophie; University Hospitals of Leicester NHS Trust<br>Harrington, Deirdre; University of Leicester College of Life Sciences, Diabetes Research Centre; University of Strathclyde |
| <b>Primary Subject Heading</b>: | Public health |
| Secondary Subject Heading: | Qualitative research, Paediatrics |
| Keywords: | PUBLIC HEALTH, PREVENTIVE MEDICINE, PAEDIATRICS |
| | |

SCHOLARONE™
Manuscripts

*I, the Submitting Author has the right to grant and does grant on behalf of all authors of the Work (as defined in the below author licence), an exclusive licence and/or a non-exclusive licence for contributions from authors who are: i) UK Crown employees; ii) where BMJ has agreed a CC-BY licence shall apply, and/or iii) in accordance with the terms applicable for US Federal Government officers or employees acting as part of their official duties; on a worldwide, perpetual, irrevocable, royalty-free basis to BMJ Publishing Group Ltd ("BMJ") its licensees and where the relevant Journal is co-owned by BMJ to the co-owners of the Journal, to publish the Work in this journal and any other BMJ products and to exploit all rights, as set out in our licence.*

*The Submitting Author accepts and understands that any supply made under these terms is made by BMJ to the Submitting Author unless you are acting as an employee on behalf of your employer or a postgraduate student of an affiliated institution which is paying any applicable article publishing charge ("APC") for Open Access articles. Where the Submitting Author wishes to make the Work available on an Open Access basis (and intends to pay the relevant APC), the terms of reuse of such Open Access shall be governed by a Creative Commons licence – details of these licences and which Creative Commons licence will apply to this Work are set out in our licence referred to above.*

*Other than as permitted in any relevant BMJ Author's Self Archiving Policies, I confirm this Work has not been accepted for publication elsewhere, is not being considered for publication elsewhere and does not duplicate material already published. I confirm all authors consent to publication of this Work and authorise the granting of this licence.*

For peer review only

# The Daily Mile in practice: Implementation and adaptation of the school running programme in a multi-ethnic city in the UK

Ash Routen[1], María González Aguado[2], Sophie O' Connell[3], Deirdre M. Harrington[4,5*]

[1] NIHR Applied Research Collaboration East Midlands, Diabetes Research Centre, Leicester General Hospital, Gwendolen Road, Leicester, LE5 4PW.

[2] School of Communication, Media and Sociology, University of Leicester

[3] University Hospitals of Leicester NHS Trust, Leicester

[4] Diabetes Research Centre, University of Leicester

[5] School of Psychological Sciences and Health, University of Strathclyde

*Corresponding author email deirdre.harrington@strath.ac.uk

**Main Body Word Count:** 5627 (excluding tables)

For peer review only

## Abstract

**Objectives:** The aim of this study was to generate new evidence on how The Daily Mile (TDM), a popular school-based running programme in the UK, is implemented in a diverse and multi-ethnic city in the UK and also the barriers faced by non-implementer schools.

**Design:** Mixed method cross-sectional study (including survey data collection and qualitative interviews).

**Setting:** Primary schools in a multi-ethnic city in the East Midlands, UK.

**Participants:** 42 schools in Leicester city completed an online survey, and five teaching staff from five schools took part in follow-up semi-structured qualitative interviews.

**Results:** Overall, 40.5% of schools who completed the survey reported having never implemented TDM, and 96.0% of implementer schools reported delivering TDM on three or more days per week. Reported barriers included space limitations and safety issues, timetabling and curriculum pressures, and pupil and teacher attitudes. Facilitators of implementation were teacher engagement and school culture/ethos, communication of the initiative and substantial delivery adaptations.

**Conclusions:** The findings from this study, based on data from schools in a multi-ethnic city in the UK, suggest that implementation of TDM is variable, and is influenced by a range of factors related to the school context, as well as the characteristics of TDM itself.

**Trial registration:** N/A


### Article Summary

### Strengths and limitations of the methods of this study

- Use of mixed methods in a multi-ethnic city.
- Views from non-implementer schools collected at two stages.
- Sample may represent schools who were particularly motivated to voice their opinion on the implementation of TDM in their school.
- The focus on one city means that these findings cannot be generalised to all primary schools.


### Funding

This work was supported by a grant via the Wellcome Trust Institutional Strategic Support Fund at the University of Leicester and funding under the *Cities Changing Diabetes* programme in Leicester. *Cities Changing Diabetes Leicester* is a joint working project that is funded by and developed in collaboration by and between Leicester Diabetes Centre and Novo Nordisk. This work has been run by researchers from the University of Leicester, Leicester Diabetes Centre and the NIHR Applied Research Collaboration East Midlands (ARC EM).

### Competing interests

None to report




For peer review only - http://bmjopen.bmj.com/site/about/guidelines.xhtml

## Background

The Daily Mile (TDM) is a physical activity programme which challenges primary schools to have their pupils run a circuit of the playground, or similar, for a minimum of 15 minutes per day during class time. Since instigation in one Scottish primary school in 2012, TDM has 6,286 (as of November 2020) registered schools and nurseries in England. A recent data linkage study reported that one in five primary English schools have registered since 2012, and that registration is higher in urban areas, and for schools with higher levels of disadvantaged pupils (1).

Support for TDM has been such that its use was advocated in schools in the 2016 and 2018 UK Government childhood obesity strategy (2, 3) and in 2017 the Scottish Parliament wrote to all schools in Scotland to promote its uptake (4). Despite policy and funding support, evidence on the TDM effectiveness and context-specific operation is only emerging. A large (2,280 children from 40 primary schools) randomised controlled trial conducted in Birmingham, England, examined effectiveness of TDM and found no significant difference (p = 0.146) in the change in the primary outcome (BMI z-score) between intervention and control schools after 12 months. The accompanying economic analysis did report TDM to be cost-effective (5).

Regional qualitative and ethnographic studies have examined how schools implement TDM and what facilitates or constrains delivery from the perspectives of head teachers, teaching staff, pupils, parents and school governors as well as stakeholders including public health practitioners. These were conducted in Scotland (6, 7), Wales (8), South London (9) and rural England (10) and represent the views of schools from a variety of deprivation and geographic locations.

These studies have identified a range of factors associated with implementation of TDM including simplicity of the intervention, flexibility of delivery, time constraints, weather and the school physical environment, impact on learning time, teacher participation, whole-school delivery, use of goal setting, adaptation and embedding into regular practice. Although a wide variety of themes have been identified, the qualitative investigations of delivery and implementation (6-10) have largely focused on perceptions of teachers and schools who currently deliver TDM. There is a gap in knowledge from schools who have chosen not to implement TDM, or those who have tried with limited success.

The aim of this evaluation was to generate new evidence on how TDM is implemented in an ethnically and socio-economically diverse city and also the barriers faced by non-implementer schools. The objectives were to describe (a) TDM adoption and reach; (b) to uncover key barriers and facilitators to TDM implementation, and (c) to identify other key issues relating to how TDM was being implemented in Leicester, UK.

## Methods

### Study design



For peer review only - http://bmjopen.bmj.com/site/about/guidelines.xhtml

We undertook a mixed method two-stage evaluation during the 2018/19 and 2019/20 academic years. Stage 1 consisted of a city-wide cross-sectional online survey, while stage 2 was an interview with primary schools who completed the first survey.

**Ethics approval**

Ethics approval was obtained from the University of Leicester College of Life Sciences Concerning Human Subjects (non-NHS) application number 19792-dh204-ls:healthsciences. A consent statement was included on the opening page of the online surveys. Individuals being interviewed provided written informed consent.

**Setting and population**

Leicester (a multi-ethnic city in the Midlands, UK) is a microcosm of the UK due to its socioeconomic diversity and proximity to suburban and rural areas. TDM in primary schools is supported by Leicester City Council strategies and schools are encouraged to sign-up via the City Council intranet and though local primary teacher meetings.

**Patient and public involvement**

This work was informed by the Public Health team at Leicester City Council, and recruitment was supported by the Leicester City School Sport and Physical Activity Network (SSPAN). No other patient or public involvement took place.

**Stage 1: City-wide survey**

The TDM co-ordinators (or PE leads) of all city primary schools were emailed by SSPAN with the initial online survey link, and the survey was also advertised on local authority schools intranet and at teacher meetings. There were eight questions on TDM implementation and experiences, if any, of TDM. For example, respondents were asked for a typical week, on how many days per week TDM is run in their school and what year groups it is delivered to. Schools reporting no delivery of TDM were asked for reasons for non-delivery. Respondents could add free text comments, and they were asked whether they would be happy to be contacted for a follow-up interview.

Stage 2 recruited schools that had adopted and utilised TDM (i.e. implementer schools), and schools that initiated TDM but had failed to maintain implementation (i.e. non-implementers).

**Stage 2: Follow-up interview**

The contact person in ‘implementer’ schools were invited via email to a face-to-face interview. In order to maximise participation of those from Stage 1 who agreed to be contacted for Stage 2 the research team phone called the school reception, send multiple emails and visited the schools within walking distance of the university in person. Interviews were conducted in schools by one trained researcher (AR) following a semi-structured topic guide. Interviews were recorded on a digital recorder and transcribed verbatim by an external transcription company. Transcripts were anonymised and identifiable information removed.

**Survey and Interview Guide**

Details of the survey and interview questions are included as supplementary files 1 and 2. The survey was designed pragmatically to gather information on the adoption of the programme, the level of implementation and any barriers to implementation. The intention



For peer review only - http://bmjopen.bmj.com/site/about/guidelines.xhtml

was to use this data to inform the support that could be offered to schools across the city by SSPAN. As such the survey was not informed by a theoretical framework. The interview questions were informed by discussion with the SSPAN and issues noted through informal conversations with local teaching staff, key themes of the RE-AIM framework such as Reach and Maintenance (11), and the programme components themselves (e.g. delivery of Daily Mile core principles).

**Analysis**

Survey data are summarised as percentages and ranges. No inferential statistical analyses were conducted on these data. A thematic analysis (12) was completed by one trained qualitative researcher (MGA); this was chosen to explore interviewees' perceptions on the implementation of the TDM at their schools. An inductive approach to analysis was followed. Transcripts were read and re-read to identify relevant features. DH supported qualitative data analysis participating in the definition and refinement of themes to enhance data reliability. Interview data were systematically coded at the descriptive and semantic level using Nvivo (version 11.4.3).

**School data**

School sociodemographic characteristics (size, geographic location, ethnic mix) were taken from School Census records (January 2019). Deprivation data of the ward (locality division for electoral and planning purposes) in which the school is situated is based on Index of Multiple Deprivation (IMD) 2019.

## Results

As of winter 2019 there were 83 primary schools available to participate in TDM in Leicester. Of these, 42 (50.6%) completed the stage 1 survey (Table 1). Of these, 19 schools responded with willingness to take part in the interview (stage 2).

**Table 1.** Characteristics of the schools involved in Stage 1 (n = 42)

| | Implementers (n = 25) | Non-implementers/ other (n = 17) |
|---|---|---|
| **Pupil number (range)** | 401 (178 – 608) | 440 (108 – 747) |
| **% BME pupils (range)** | 49.8 (21.5 – 97.8) | 71.5 (20.2 – 98.0) |
| **% FSM (range)** | 23.4 (5.1 – 44.9) | 17.9 (108 – 758) |
| **% in IMD Q1 (range)** | 40.0 (0 – 100) | 24.5 (0 – 61.1) |
| **% in IDACI Q1 (range)** | 48.9 (10.8 – 100) | 35.7 (9.2 – 69.6) |

Note: BME – black and minority ethnicity; FMS – free school meals; IMD Q1 = quartile one (most deprived) of Index of Multiple Deprivation; IDACI Q1 **-** Income Deprivation Affecting Children Index Quartile one (most deprived).

***Implementation level and type***



For peer review only - http://bmjopen.bmj.com/site/about/guidelines.xhtml

For peer review only

Of the schools that completed the survey, 40.5% (17/42) reported never running TDM. Of those who did implement it (i.e. 59.5%; 25/42), 56.0% (14/25) reported running it five days/week, 4.0% (1/25) on four days, 36.0% (9/25) on three days, and 4.0% (1/25) on two days/week.

Table 2 presents the proportions of schools delivering TDM to various combinations of year groups. For example, 69% (20/29) of schools implementing TDM deliver it to all year groups from foundation year, to year 6. Schools reported in the survey that TDM delivery "*varies for different year groups*" and the "*amount actually varies greatly between classes and teachers.*"

**Table 2.** Proportions of schools delivering TDM to year groups

| Year groups | Foundation | Year 1 | Year 2 | Year 3 | Year 4 | Year 5 | Year 6 |
|---|---|---|---|---|---|---|---|
| % | 71.9 | 75.0 | 81.3 | 87.5 | 90.6 | 87.5 | 87.5 |

The survey free text responses provided relevant insight on the schools' reasons for not delivering TDM. Four participants reported that their schools were actually implementing an adaptation of TDM: "*We do a less formal version where classes go for a walk, jog about three times per week*". On the question "Why does your school not deliver TDM?" eight participants reported difficulty in delivery and/or resource or staffing problems. One school mentioned parents' lack of support to the initiative as a reason not to deliver TDM.

***Stage 2 - Qualitative themes***

Interviews were conducted with TDM coordinators at five primary schools (see school characteristics in Table 3). Two of the five schools either did not deliver TDM or had previously delivered and discontinued after a few months. One of the schools delivered TDM before school at a local cricket ground (which they pay for) and parents sometimes watch the children.

**Table 3.** Characteristics of the schools involved in Stage 2

| School | School pupil number | % BAME | % FSM | Deprivation of ward | TDM Delivery | Contact person's role within school |
|---|---|---|---|---|---|---|
| 1 | 596 | 51.5 | 7.4 | Quintile 4 | No, "it was done sporadically for a few months" | Higher level teaching assistant and PE co-ordinator |
| 2 | 379 | 59.4 | 25.3 | Quintile 1 | Yes | Senior management team |
| 3 | 366 | 24.6 | 25.1 | Quintile 1 | Yes | PE co-ordinator and class teacher |


For peer review only - http://bmjopen.bmj.com/site/about/guidelines.xhtml

| | | | | | | |
|---|---|---|---|---|---|---|
| 4 | 235 | 49.8 | 20.4 | Quintile 2 | No, The Daily Boost as an alternative is on-going | PE and history co-ordinator and class teacher |
| 5 | 468 | 40.6 | 23.4 | Quintile 2 | Yes, although The Daily Boost is used with years 1 and 2 | PE Teacher |

Note: Deprivation of ward is based on IMD where a lower quintile is the most deprived; BAME = Black, Asian and Minority Ethnic group; FSM = free school meals.

Themes emerged through the aggrupation of representative subthemes that reflected the research questions, as well as new emergent features in the data. The iterative comparison of the responses in the interviews resulted in a coding framework consisting of five themes: 'Barriers to the delivery of TDM', 'facilitators of the TDM', 'tailoring physical activity offerings', 'perceived benefits' and 'measuring TDM success.' The description of these themes and the content of sub-themes are below.

## 1) Barriers to the delivery of TDM

### 1.1 Characteristics and functioning of the school

Three types of barriers related to the characteristics and functioning of the school were identified: space and safety concerns, pressures to deliver the National Curriculum and timetabling difficulties.

Four of the five interviewees affirmed that the lack of a safe and appropriately sized playground or field complicates the running of TDM, while three reported not having adequate space for the optimal running of TDM. Safety concerns relating to the quality of the space where pupils run also emerged as a possible implementation barrier. These concerns were related to the safety of the playground during adverse weather (for instance, puddles or icy pavement), that some pupils from disadvantaged families don't have appropriate footwear for all weather conditions and the risk of having a high number of pupils running at the same time in a small playground:

> *"(…) obviously, if we took a few classes down at a time health and safety becomes a little bit of an issue because we've got benches in the way, we've got bits around the playground so trying to get the boys not to be racing round and the girls."*
>
> (School 1)

The description of space as an important barrier resonates with the free text qualitative data from non-implementer schools in Stage 1. These schools reported lack of space as one of the primary reasons for not delivering TDM. Some schools, especially those with larger pupil numbers, feel that they do not have enough space to deliver TDM: *"Due to the space and the size of the school. We only have a playground and 480 children. When PE is on there is a lack of space due to safety."*

A second barrier within this theme is the teachers/pupil timetables. Four schools found it difficult to fit TDM in their timetables without negatively impacting other teaching duties, such



For peer review only - http://bmjopen.bmj.com/site/about/guidelines.xhtml

as assessments and lessons. In addition, delivering TDM on a daily basis makes one of the lessons fifteen minutes shorter.

> *"Well, in a week where you just think ahh, I've got so much to get through and we spent longer doing this so now we're running behind and it feels like that a lot of the time for a lot of us that we're always trying to fit more in than really we can. So, for some people. The Daily Mile is a thing too far, sadly."*
>
> (School 2)

Stage 1 participants also highlighted the difficulties including TDM in the school's timetable as a barrier to delivery: "*Teachers timetables are so full with regular lessons and other initiatives that The Daily Mile would be too much of a burden to ask them to fit as well*" and "*They [pupils] already do a lot, don't know where it would go in the timetable*."

The third barrier within this theme was the pressure to deliver the National Curriculum. TDM coordinators intimated that delivering the curriculum is more important than pupils' daily physical activity, which could constrain TDM implementation. But it is important to note that two interviewees contend that TDM's impact on curriculum delivery is minimal, as having a physical activity break helps pupils focus. The excerpt below expresses interviewees' perception that the pressure to deliver the curriculum might be a barrier to the adoption and implementation of TDM:

> *"Even four- year olds are being baselined now [inaudible] timetables and all of this is like ways in which school will be held accountable by Ofsted [inaudible]. So, things like The Daily Mile go on the backburner, don't they? Which is a shame."*
>
> (School 4)

**1.2 Pupils' and teachers' attitudes**

The second type of barrier related to pupils' and teachers' attitudes towards physical activity, the outdoors and TDM. Interviewees considered that teachers' personal attitudes towards physical activity and being outdoors influences the implementation of TDM:

> *"I think that is the biggest barrier* [school's space] *and then some people's lack of enthusiasm for being outside and being active which you get quite…if you have a range of staff that's what you're going to get"*
>
> (School 2)

**1.3 TDM in practice**

The main sub-theme was that TDM is "not as simple" in practice. Although interviewees agreed that the TDM is easy to implement, three affirmed that it is not exempt of costs and implementation difficulties. They suggested TDM takes more than fifteen minutes, and involves additional costs for TDM to run autonomously. As outlined in TDM core principles (13), the TDM has to be quick to deliver. However, the estimated fifteen minute delivery time does not include time needed to shepherd pupils to the playground, especially if the classroom lacks direct access to the open area. One teacher suggested the real implementation time can be up to thirty minutes.



For peer review only - http://bmjopen.bmj.com/site/about/guidelines.xhtml

Running TDM may involve additional costs. Here, interviewees were not referring to small expenses, such as footprints (painted on the playground to guide children) or tokens to count laps, but to staffing costs and teachers' time after work for additional preparation that optimal, engaging and long-term implementation requires. Interviewees explained that they invested time outside their working hours to collate pupils' times or create pupil progress certificates. One of the interviewees reported that TDM delivery required the collaboration and practical support of numerous teachers and other school staff:

> *"It does need managing so we have members of staff out. So, dinner ladies, lunch time supervisors and the class teachers now come out to manage because people can get into little groups and cause little bits of aggro so we try and nip that in the bud as much as possible."*
>
> (School 5)

**2) Facilitators of TDM delivery**

**2.1 Schools' existing health and sport culture/ethos and teacher motivation**

The approach of the school to health and physical activity can facilitate the long-term success of TDM in a school. All interviewees agreed that the TDM builds into, and contributes to, the wider culture or ethos of health and wellbeing within the school. Four interviewees described TDM as part of the school's health and sports culture, as an element of the "school's values":

> *"Yeah, part of our values is that actually being active and healthy is important and I think we do promote being healthy, our children get an opportunity to do cooking which they don't in a lot of schools, which is great, and we promote healthy eating through that but I think just being active, encouraging that would be, yeah, for it* [TDM] *to be part of our school values would be good if it was a greater part of our school values."*
>
> (School 2)

Interviewees mentioned several times the importance of the support from the school in running TDM. By support from the school, interviewees referred to the schools' interest in introducing TDM and the acknowledgment that pupils' daily physical activity and the TDM are an important part of the school's values and activities. Teachers' motivation and the development of a health and sports culture within the school can positively influence TDM delivery. Interviews responses showed that teachers' involvement in the delivery of TDM impacts pupils' performance. Teachers running alongside and cheering pupils was reported to be a motivator and aid for pupil engagement.

**2.2 Communication of TDM**

Interviews revealed that communicating the TDM is a key to facilitating implementation. Schools make use of their communication channels (e.g. newsletters), to communicate that they are running TDM. For some schools, the school's weekly assembly is a key communication space to engage students with TDM and reinforce the role of TDM as part of the school's health culture. During the school assembly, for instance, weekly results of TDM competition (see 2.4 below) are communicated and progress certificates handed out:



For peer review only - http://bmjopen.bmj.com/site/about/guidelines.xhtml

> *"(…) if they're doing the right thing then they'll keep going and some children do eight laps, some children do two and at the end of the week I record it all, put it on a spreadsheet so I've got the data of who is doing what, when. We look at celebrating that in assembly with the class that goes the furthest."*
>
> (School 5)

### 2.3 Ability to modify their limited space

Interviewees explained that small modifications in playgrounds to mark the path or giving pupils a token to count laps can facilitate TDM. One interviewee reported that her school have painted footprints on the playground to make running TDM more intuitive. Interviewees regard the use of tokens and clickers to count laps as positive:

> *"Yes, something I hadn't thought of, but think could be quite useful, sometimes if children could have clickers, you know when they count people into things, so if they could click every time, they do a lap that might be good in terms of resources."*
>
> (School 2)

### 2.4 "Keep it fresh"

As noted above, if schools deliver TDM according to the core principle of simplicity (getting pupils to run or walk for fifteen minutes per day) pupils "*would lose interest in it after a week quite quickly*." All the interviewees explained that they had to introduce some variations or adapt TDM to maintain pupils' engagement with the initiative in the long-term. Coordinators developed diverse strategies to maintain pupils' motivation and interest in TDM. Strategies included introducing an element of competition (inter class competitions), monitoring and rewarding pupils' progress and "*keeping the TDM fresh*". As one interviewee explains, they made TDM complicated to engage pupils:

> *"I've made it complicated to suit what I want to get out of it. If I want my children to do something I have to have that element of competition (…) So yeah, I'd agree with that and I just make a rod for my own back to make it more enjoyable for the children, I guess. For my children because a sleepy village in Scotland has a different cohort to inner city Leicester."*
>
> (School 5)

Monitoring pupils' times and distance ran was regarded as motivating for pupils by coordinators. It is necessary to clarify that that monitoring does not aim at promoting individual competition or creating an "elite" group of fitter pupils, but to encouraging self-improvement by presenting TDM as a collective endeavour. Regarding competitivity among pupils it is important to note that the school 1 interviewee contended that competition does not always work, as many pupils are not competitive.

To ensure pupils' interest and engagement with TDM in the long-term, interviewee number 5 explained that teachers and schools have to 'keep TDM fresh' to motivate and engage pupils. 'Keep TDM fresh', in this case, means updating the initiative on an annual basis, so pupils maintain their interest and motivation year after year. "*Keeping TDM fresh*" requires modifying the initiative at two levels: 1) Introducing changes in the TDM implementation design for the academic year (i.e. measuring the accumulative time and translating into distance on a map, i.e. "*this year [we] have run the distance between Spain and the UK*"; introducing a system of houses, pupils run for their house to win etc.) and 2) measures to implement TDM on a daily basis (i.e. monitoring pupils time, use of tokens, mark the distance pupils have to run).



For peer review only - http://bmjopen.bmj.com/site/about/guidelines.xhtml

### 3) Tailoring their physical activity offering

Schools are interested in delivering physical activity programmes to keep their pupils active daily. Schools adapted programmes to overcome internal barriers and engage pupils. Four of the five interviewed schools combine different daily exercise programmes overcoming spatial, timetable and curriculum barriers.

Two interviewees introduced two alternative physical activities to deliver instead of TDM in adverse weather in the absence of a covered playground. Another school used different physical activity initiatives in different school years according to pupils' age to create a culture of health and sports in the school. In this school, younger pupils do The Daily Boost - an initiative encouraging fifteen minutes of organised physical activity at the school, this can be running, walking, cycling, dancing or any physical activity pupils may like - while TDM is usually implemented among the older pupils, so younger pupils associate transitioning to higher years with running TDM:

> *"Oh yeah, so we rolled out The Daily Boost to kind of…so The Daily Mile was running quite happily and then we've gone well, we need to do something for the younger children to get them. So, it's not just a shock at Year 3, so it slowly integrates into their school life. So yeah, we have started to roll that out and that was rolled out at the start of this year."*
>
> (Interview, 5)

Schools, hence, create their "*own version*" of the TDM to successfully implement the TDM according to the necessities and characteristics of their schools and pupils:

> *"Yes, so we've created our own version to keep the children engaged. You see with the skipping, there's so many different variations. They can learn tricks; they can do spins. Like we've got a few big ropes so they're doing different games, different activities with the big ropes, they just love having little competitions against each other to see who can skip for the longest, it's just a little bit more, they can vary it so much whereas with the Daily Mile you can't really, there's not that much to vary apart from saying to them let's see if you can increase your time but again."*
>
> (School 1)

The interview findings reflect the Stage 1 survey reporting that schools, despite the mentioned barriers, try to develop strategies to help their situation. For instance, by applying for funds to improve their space as in the excerpt *"Grant applied for to fund a track"* or to ensure their pupils could still be active in a similar fifteen-minute window using skipping ropes. Schools, in particular, were implementing The Daily Boost which is seen as more practical and offered flexibility for their size and space.

### 4) Perceived benefits of TDM

All interviewees reported that TDM benefits their pupils. The benefits can be classified into physical and behavioural. For physical it is through the improvement in fitness or by encouraging them to practice sports outside the school. Two teachers reported that TDM improved the physical condition of their pupils and increased their physical activity levels.

Interviewees 1 and 5 explained that TDM acted as a catalyser of pupils like for being physically active and sports and making healthier choices. Interviewee 5 tells that one pupil started to



For peer review only - http://bmjopen.bmj.com/site/about/guidelines.xhtml

become interested in practising sports after school as a consequence of the implementation of TDM at the school:

> *"So, I've got one family who she started doing cross country in Year 3, she's won the medal, so now she goes running with her mum and I didn't expect that to happen and now she loves it, she loves running and she's been successful at it and it's kind of given her an into other sports. Now she's part of the girls' football team, she plays cricket, she gets stuck in with everything, and the running was the catalyst."*
>
> (School 5)

The interviewees agreed in that TDM not only have physical benefits for pupils, but it also brings about behavioural and social benefits. Four interviewees agreed in that that running TDM improved pupils' attention and concentration. As they explained, having a break for physical exercise help them re-focus on their learning. Interviewee 1 also affirmed that TDM help pupils to manage their anger:

> "(…) we have a few children that perhaps have behavioural problems, anger problems and you know, they go out and it does help them. They'll say to you that it helps them, and it helps them focus a little bit more if they have that break, go out, do that, come back in and they feel that…"
>
> (School 1)

### 5) Measuring success

Although interviewees reported the TDM to be beneficial, there is no evidence of the benefits of the TDM beyond teachers' own perceptions. The interviewees' focused on "keeping pupils active" putting the ways in which pupils do TDM in a secondary place. The interviewees described TDM as beneficial without giving excessive importance to the fact that some pupils (especially girls) walk instead of running or if they run in smaller laps if the playground is too busy. Interviewees described TDM as a successful initiative because it helps them to ensure that pupils are physically active ("The whole thing is keeping them active"):

> *"The most important thing for me is that it's done. If we're really squeezed for time and to be honest, sometimes we are and I'll say right, we're going to do half a mile."*
>
> (School 2)

## Discussion

### Main findings

Over half of schools in our survey have adoption of TDM in some form while40.5% who completed the survey reported never implementing TDM. In relation to dose delivered, 56.0% of schools who adopted TDM ran TDM every day which was a TDM recommendation at time of data collection. Subsequently TDM recommend delivery on at least three days, which nearly all schools (96.6%) met. The majority (69%) report delivering TDM to all year



For peer review only - http://bmjopen.bmj.com/site/about/guidelines.xhtml

groups from foundation year, to Year 6, suggesting there is good reach across the school. However, teachers acknowledged delivery varies within and across year groups.

Our results from an ethnically and socioeconomically diverse city make a useful addition to the extant literature. Although it was highlighted that what works in a rural area in Scotland would not work for a multi-ethnic city like Leicester no themes related to ethnicity emerged in our study. However, Stage 1 data show the percentage of schools in the lowest deprivation quartile was higher in the implementer than non-implementer schools which supplements data showing that TDM-registered schools had more disadvantaged pupils (1).

TDM coordinators conveyed a range of class, teacher and organisational level issues they felt constrained the delivery of TDM including space limitations and safety concerns, national curriculum pressures, and timetabling difficulties. These themes shed light on how TDM core principles (13) of TDM being quick and kept simple may not always be possible. In relation to pupils and teachers, attitudes toward physical activity and the outdoors appeared to act as a constraint to implementation. Facilitators of delivery included coordinating teachers' motivation, and the existing school ethos and culture regarding physical activity and wellbeing. Finally, the need to adapt the delivery of TDM to maintain pupil motivation was identified as an important issue, as was the creation of alternative physical activity programmes, to supplement TDM, or to deliver in place of TDM, due to the needs of the given school context.

## Strengths and limitations of the study

Strengths of this study include the use of mixed methods, with the survey providing information on the implementation of TDM across a wide range of schools in a multi-ethnic city. Only one extant study has examined TDM implementation at a city/borough level (9). This enabled identification of variation in practice, including schools who did not implement TDM, which was further examined by following-up a small number of schools for more detailed understanding via interviews. A limitation of this approach is that our interview sample may represent schools who were particularly motivated (schools self-selected availability for inclusion in further research in the survey) to voice their opinion on implementation of TDM in their school i.e. successful implementer schools, or conversely those with strongly held views on the difficulties or non-implementation. A limitation is the response rate of 50% for the online survey which could introduce non-response bias. Difficulties in accessing school staff is not uncommon (14) and practical aspects, such as limited availability of respondents, are explanations for limited sample sizes (15). The interviews provided rich and nuanced codes and "meaning saturation" (16) was achieved. The obtained data met additional quality criteria in qualitative research (17): codes were supported by a wide range of evidence from data and findings resonated with the literature. Therefore, we do not believe the number of interviews affects the validity or generalisability of our findings (15). However, as is inherent within qualitative work, and the focus on a densely populated urban area, our findings cannot be generalised to all primary schools.

## Comparison to existing literature

The adoption rate of 59.5% is slightly higher than the 48% reported from a TDM evaluation in London schools (9). However, our value is based on the survey respondents only so it is likely this number is lower. Two previous studies have detailed the dose of TDM delivered in schools. Focused on one primary school in the East Midlands, Harris et al. (10) reported key stage teachers delivered TDM on 86% of days, with 95% of pupils receiving the intervention on those days. Hanckel et al. (9) qualitatively summarised dose information, suggesting TDM was not delivered every day in most schools/classes, and that it was viewed as interchangeable with other physical health interventions within the school. To our knowledge there is no other existing data on reach of TDM delivery across year groups.


For peer review only - http://bmjopen.bmj.com/site/about/guidelines.xhtml

The barriers of space limitations, timetabling and pressures to deliver the curriculum are consistent with findings from previous TDM evaluations (7-9, 18) as well as being common barriers to the delivery of other physical activity initiatives or policies in schools (19, 20). Likewise teacher buy-in (i.e. whether they thought it worthwhile and whether they liked it or not) has been reported as a barrier in other school-based running programmes (21). In relation to implementation facilitators, previous work on TDM has identified that teacher's active participation in TDM is important for enabling pupil engagement with the initiative (8) The importance of TDM aligning with the existing school culture and ethos towards physical activity and wellbeing, and therefore acting as a facilitator to implementation, has been highlighted in research on a similar school-based running programme in the UK (21). Schools' substantial adaptations of the TDM and the development of rebranded, alternative physical activity offerings resonate with a recent qualitative study that highlighted the malleability of TDM (18). Adapting in an effort to counteract a drop off in activity levels (22) and to respond to the need to keep pupils engaged and reduce boredom though offering variety or choice.

## Implications for practice

It is clear that adaptations and alternatives to TDM are happening. This has implications for TDM evidence base as well as practice. Schools may begin using TDM but adapt or change their approach, often quite substantially, to suit their particular context. This has implications for the relevance of any evidence for effectiveness of TDM in a real-world setting, as some schools may not necessarily be delivering TDM even if using TDM nomenclature. When promoting TDM to diverse schools within a local authority area, strategies to support schools appropriately adapt TDM or even create alternatives to keep pupils active for fifteen minutes according to the needs and preferences of their school are needed. We didn't find a need to adapt based on ethnic make-up of the school. One unique point related to disadvantage emerged whereby pupils' shoes were not suitable for all weathers. While a TDM core principle is that children should not need specialist kit and can do it in their school clothes it is conceivable that children in light or low-quality shoes may find their mile on wet tarmac uncomfortable. Equally, TDM risk assessments state that children in unsuitable footwear should walk (23) which also has implications for the pupil's own experience and when evaluating the TMD at the pupil level as there are different health and social effects to running and walking a mile. It is important that schools and stakeholders consider factors that support long-term implementation prior to starting TDM in a school. In particular consideration should be given to the existing implementation context within the school, such as the physical environment (space, playground options) organisational issues (timetabling, curriculum focus/pressure) staffing (teacher attitudes and motivation), as well as the schools culture and ethos regarding physical activity and health. These considerations are also relevant to any physical activity programme that is embedded in schools. It may also be important to emphasise to schools that in spite of apparent simplicity, TDM does require teacher time and additional effort to maintain pupil interest and engagement, and it may involve a collective effort involving staff from across the school until it can run autonomously. Finally, as is the case with Leicester city schools, it is clear there are ways to adapt TDM to facilitate delivery and allow children to participate in a vital fifteen minutes of physical activity that it distinct from PE and embedded in curriculum time.

## Conclusions and future research

The findings from this study, based on data from schools in a multi-ethnic city in the UK, suggest that implementation of TDM is variable, and is influenced by a range of factors related to the school context, as well as the characteristics of TDM itself. Future research should focus on understanding how to support schools in readiness for implementation of TDM and school-based running programmes, and learning from schools that implement adapted versions of the TDM, or alternative programmes with similar core principles.



For peer review only - http://bmjopen.bmj.com/site/about/guidelines.xhtml

## References

1. Venkatraman T, Honeyford K, Costelloe CE, Ram B, M F van Sluijs E, Viner RM, et al. Sociodemographic profiles, educational attainment and physical activity associated with The Daily Mile™ registration in primary schools in England: a national cross-sectional linkage study. Journal of Epidemiology and Community Health. 2020:jech-2020-214203.
2. UK Government. Childhood Obesity Strategy: A Plan for Action. 2016.
3. UK Government. Childhood Obesity Strategy: A Plan for Action, Chapter 2. 2018.
4. Scottish Government. Scotland: a Daily Mile Nation. 2017.
5. Breheny K, Passmore S, Adab P, Martin J, Hemming K, Lancashire ER, et al. Effectiveness and cost-effectiveness of The Daily Mile on childhood weight outcomes and wellbeing: a cluster randomised controlled trial. International Journal of Obesity. 2020:1-11.
6. Ryde GC, Booth JN, Brooks NE, Chesham RA, Moran CN, Gorely T. The Daily Mile: What factors are associated with its implementation success? PloS one. 2018;13(10).
7. Malden S, Doi L. The Daily Mile: teachers' perspectives of the barriers and facilitators to the delivery of a school-based physical activity intervention. BMJ open. 2019;9(3):e027169.
8. Marchant E, Todd C, Stratton G, Brophy S. The Daily Mile: Whole-school recommendations for implementation and sustainability. A mixed-methods study. PloS one. 2020;15(2):e0228149.
9. Hanckel B, Ruta D, Scott G, Peacock JL, Green J. The Daily Mile as a public health intervention: a rapid ethnographic assessment of uptake and implementation in South London, UK. BMC public health. 2019;19(1):1167.
10. Harris J, Milnes LJ, Mountain G. How 'The Daily Mile™' works in practice: A process evaluation in a UK primary school. Journal of child health care. 2019:1367493519880049.
11. Glasgow R, Boles S, Vogt T. RE-AIM framework [Available from: https://www.re-aim.org/about/what-is-re-aim/.
12. Braun V, Clarke V. Using thematic analysis in psychology. Qualitative research in psychology. 2006;3(2):77-101.
13. The Daily Mile Foundation. 10 core principles. Your steps to Daily Mile success 2020 [Available from: https://thedailymile.co.uk/wp-content/uploads/2020/07/A4-Core-Principles.pdf.
14. Oates C, Riaz NN. Accessing the field: Methodological difficulties of research in schools. Education in the North. 2016;23(2):53-74.
15. Vasileiou K, Barnett J, Thorpe S, Young T. Characterising and justifying sample size sufficiency in interview-based studies: systematic analysis of qualitative health research over a 15-year period. BMC Med Res Methodol. 2018;18(1):148.
16. Hennink MM, Kaiser BN, Marconi VC. Code saturation versus meaning saturation: how many interviews are enough? Qualitative health research. 2017;27(4):591-608.
17. Nelson J. Using conceptual depth criteria: addressing the challenge of reaching saturation in qualitative research. Qualitative research. 2017;17(5):554-70.
18. Ward G, Scott D. Negotiating the Daily Mile Challenge; looking-like a walking break from the classroom. Sport, Education and Society. 2021;26(2):119-34.
19. Nathan N, Elton B, Babic M, McCarthy N, Sutherland R, Presseau J, et al. Barriers and facilitators to the implementation of physical activity policies in schools: a systematic review. Preventive Medicine. 2018;107:45-53.
20. Routen AC, Johnston JP, Glazebrook C, Sherar LB. Teacher perceptions on the delivery and implementation of movement integration strategies: the CLASS PAL (physically active learning) Programme. International Journal of Educational Research. 2018;88:48-59.
21. Chalkley AE, Routen AC, Harris JP, Cale LA, Gorely T, Sherar LB. A retrospective qualitative evaluation of barriers and facilitators to the implementation of a school-based running programme. BMC Public Health. 2018;18(1):1189.


For peer review only - http://bmjopen.bmj.com/site/about/guidelines.xhtml

22. McGann J, Meegan S, Murtagh E, Duff C, Belton S. "… The Way That You Do It": An Exploratory Study Investigating a Process-versus Outcome-Oriented Approach to School-Based Physical Activity Promotion. Advances in Physical Education. 2020;10(3):262-81.
23. The Daily Mile Foundation. Sample risk assessment 2020 [Available from: https://thedailymile.co.uk/resources/.

**Acknowledgements**

We thanks Daxa Ralhan from Leicester City Council and Sarah Lansdowne from Leicester City School Sport and Physical Activity Network for assisting with school recruitment. We thank Andrew Fry and Anna Harding from the University of Leicester for administering the funding.

**Author contribution statement**

AR, DH and SC conceived the original study design and methods. AR collected data and MGA led the qualitative analysis. MGA and DH discussed and refined the emerging codes and final themes. AR, DH and MGA wrote the first draft of the manuscript. All authors reviewed the manuscript and provided feedback and comments. All authors read and approved the final manuscript.

**Data sharing statement**

Data sharing is not possible as it was not included in the consent forms. However, all reasonable requests for access the data for reanalysis will be considered. Please contact the corresponding author.

For peer review only

UNIVERSITY OF LEICESTER

# Daily Mile Survey

## Welcome

The Daily Mile is being used in schools all over the UK. A team of researchers at the University of Leicester are doing some research on the Daily Mile in Leicester City.

The first step of this research is to find out how the Daily Mile is used in schools once they have signed up to the National Website.

This short survey is about the Daily Mile at your school.

Even if you do not use the Daily Mile at your school we would appreciate you answering some brief questions as to why it is not used in your school.

At the end, you can decide whether your school can be contacted by the research team from the University of Leicester regarding the second stage of the project.

For peer review only


For peer review only - http://bmjopen.bmj.com/site/about/guidelines.xhtml

# Background

What is the name of your school?

Has your school signed up for the Daily Mile via the National Website?

- Yes
- No

For a typical week, on how many days per week is the Daily Mile run in your school?

- Never
- 1
- 2
- 3
- 4
- 5
- Other

Other (please comment):



For peer review only - http://bmjopen.bmj.com/site/about/guidelines.xhtml

# Delivery

Why does your school not deliver the Daily Mile? Please select all that apply.

- ☐ Financial/funding reasons
- ☐ No support from teachers
- ☐ No support from parents
- ☐ Children already taking part in another active mile initiative
- ☐ Resource/staffing problems
- ☐ Difficulty in delivery
- ☐ Other

Difficulty in delivery (please comment):

Other (please comment):

Would your school benefit from some help or advice (from the Leicester City School Sport and Physical Activity Network) to deliver the Daily Mile?

- ○ Yes
- ○ No


For peer review only - http://bmjopen.bmj.com/site/about/guidelines.xhtml

# Delivery

Does your school deliver the Daily Mile to all year groups?

- ○ Yes
- ○ No

For peer review only

# Participation

Please select all applicable year groups that take part in the Daily Mile in your school.

- ☐ Foundation
- ☐ Year 1
- ☐ Year 2
- ☐ Year 3
- ☐ Year 4
- ☐ Year 5
- ☐ Year 6

For peer review only

# Next Steps

If you would like to get involved in a research study by the University of Leicester about the Daily Mile please add your email address here or add the contact email of another staff member who can answer questions about the Daily Mile at your school.

For peer review only


For peer review only - http://bmjopen.bmj.com/site/about/guidelines.xhtml

# Submit your answers

Please click 'Finish' to submit your answers.

For peer review only

# Thank you!

Thank you for taking part in this short survey.

If you provided contact details we will be in touch soon regarding the next stage of the research.

For peer review only


For peer review only - http://bmjopen.bmj.com/site/about/guidelines.xhtml

**Example: Daily Mile Evaluation Coordinator/Head teacher Interview Guide**

| | | |
|---|---|---|
| **Name of Teacher**: | **Date**: | **Start time**: |
| **School**: | | **End Time**: |

| | Questions | To Probe | Checklist |
|---|---|---|---|
| **1** | **Reach** | | |
| a) | How did you first hear about the Daily Mile? | Who are the key players, what steps had to be taken at the school to get buy-in. | |
| b) | How did you hear about the opportunity to sign up locally? | | |
| c) | Why did you decide to sign your school up to the Daily Mile? | | |
| d) | Describe the process of getting the school on board for this | | |
| **2.** | **Level of Implementation** | | |
| a) | Can you tell me how the Daily Mile is being run in your school? | Frequency and Duration | |
| **3.** | **Processes of Implementation [MISS OUT FOR NON-IMPLEMENTER & HEAD]** | | |
| a) | How do the pupils move when they do the Daily Mile? | Walk, run, skip, hop, hold hands etc. | |
| b) | Where do you deliver the Daily Mile? | Playground, field, other site. | |
| b) | Please can you talk me through how you have approached each of the core DM principles in your school? | Show print out of DM principles. Adaptations e.g. skipping the DM, using non-curricular time | |
| c) | What are the practical preparations that you have done to deliver DM? | Clothing, weather, staffing, space. | |
| d) | How do you monitor pupil participation and pupil progress?<br>How do you use the Daily Mile in the teaching of any curriculum lessons? | Attendance, lap counting. | |



For peer review only - http://bmjopen.bmj.com/site/about/guidelines.xhtml

| | Questions | To Probe | Checklist |
|---|---|---|---|
| | Tell me about any adaptations you had to make to the school timetable in any way to deliver DM? | | |
| e) | Beyond normal teaching time, tell me about any additional staff time or additional resources that have been used to deliver DM. | | |
| 4 | **Implementation Drivers** | | |
| a) | Tell me about any things which have made it difficult to deliver the Daily Mile in your school? (Barriers) | Pupil, teacher and school level issues. E.g. lack of staff to supervise, pupil disinterest, no SMT support. | |
| b) | Tell me about any things which have made it easier to deliver the Daily Mile in your school? (Facilitator) | | |
| 5 | **Perceived Outcomes [MISS OUT FOR NON-IMPLEMENTER]** | | |
| a) | Tell me about any changes in your pupils since doing the Daily Mile? | Positive changes such as improved attention in class, fitness etc. | |
| b) | Tell me about any changes in your teachers since doing the Daily Mile? | Negative changes such as misbehaviour in class. | |
| 7 | **Unintended Consequences** | | |
| a) | Some schools have reported things changing in their schools that they were not expecting after starting TDM. Is there anything you can think of that changed? | We have examples from other schools. Let me tell you about them.<br>a. Other programmes have been stopped because TDM has started.<br>b. School decision makers are looking more favourably on physical activity within the school.<br>c. Time used for PE is being dedicated to TDM instead. | |
| b) | Think firstly for the pupils. Think then for the teachers. Think then for the school decision makers. | | |
| 8 | **Maintenance [MISS OUT FOR NON-IMPLEMENTER]** | | |



For peer review only - http://bmjopen.bmj.com/site/about/guidelines.xhtml

| | Questions | To Probe | Checklist |
|---|---|---|---|
| a) | Can you tell me how much or if the DM is now part of the school culture at your school? | Extent of routine practice, community involvement (parents), leadership support, school policy etc. | |
| b) | Can you describe what will/or is your school doing to continue the Daily Mile in future | School policy, funding, external support etc. | |
| **9** | **Decision to stop [NON-IMPLEMENTER ONLY]** | | |
| a) | Why did you decide not to start the Daily Mile in your school? | Probe pupil, teacher and pupil barriers as above. | |
| b) | Why did you decide to stop delivering the Daily Mile in your school? | Probe pupil, teacher and pupil barriers as above. | |
| **10** | **Additional Questions** | | |
| a) | How would you describe your role within the Daily Mile at your school (are they an intervention agent?) | | |
| b) | Tell me what support you have looked for/been offered/received. Is there anything more you would need? | | |
| c) | The Daily Mile has been called “simple”. What do you think about that statement? | Set-up and maintenance; if anything is thought to not be simple follow-up on that. | |
| d) | You said that XXXX was not simple/a challenge, how have you overcome that issue? Anything else? | | |
| e) | The Daily Mile has been called “inexpensive”. What do you think about that statement? | Set-up and maintenance; if anything is thought to cost follow-up on that. | |
| f) | What do you think is ‘success’ in terms of delivering the Daily Mile in your school? | | |
| Is there anything else we haven’t already mentioned or talked about which you think would be useful to know? Do you have any questions? | | | |

# Reporting checklist for cross sectional study.

Based on the STROBE cross sectional guidelines.

## Instructions to authors

Complete this checklist by entering the page numbers from your manuscript where readers will find each of the items listed below.

Your article may not currently address all the items on the checklist. Please modify your text to include the missing information. If you are certain that an item does not apply, please write "n/a" and provide a short explanation.

Upload your completed checklist as an extra file when you submit to a journal.

In your methods section, say that you used the STROBE cross sectionalreporting guidelines, and cite them as:

von Elm E, Altman DG, Egger M, Pocock SJ, Gotzsche PC, Vandenbroucke JP. The Strengthening the Reporting of Observational Studies in Epidemiology (STROBE) Statement: guidelines for reporting observational studies.

For peer review only

| | | Reporting Item | Page Number |
|---|---|---|---|
| **Title and abstract** | | | |
| Title | #1a | Indicate the study’s design with a commonly used term in the title or the abstract | 2 |

| | | | |
|---|---|---|---|
| Abstract | #1b | Provide in the abstract an informative and balanced summary of what was done and what was found | 2 |
| **Introduction** | | | |
| Background / rationale | #2 | Explain the scientific background and rationale for the investigation being reported | 3 |
| Objectives | #3 | State specific objectives, including any prespecified hypotheses | 3 |
| **Methods** | | | |
| Study design | #4 | Present key elements of study design early in the paper | 3 |
| Setting | #5 | Describe the setting, locations, and relevant dates, including periods of recruitment, exposure, follow-up, and data collection | 3 |
| Eligibility criteria | #6a | Give the eligibility criteria, and the sources and methods of selection of participants. | 4 |
| | #7 | Clearly define all outcomes, exposures, predictors, potential confounders, and effect modifiers. Give diagnostic criteria, if applicable | N/A |
| Data sources / measurement | #8 | For each variable of interest give sources of data and details of methods of assessment (measurement). Describe comparability of assessment methods if there is more than one group. Give information separately for for exposed and unexposed groups if applicable. | 4 |

For peer review only

| | | | |
|---|---|---|---|
| Bias | #9 | Describe any efforts to address potential sources of bias | N/A |
| Study size | #10 | Explain how the study size was arrived at | 4 |
| Quantitative variables | #11 | Explain how quantitative variables were handled in the analyses. If applicable, describe which groupings were chosen, and why | 4 |
| Statistical methods | #12a | Describe all statistical methods, including those used to control for confounding | N/A |
| Statistical methods | #12b | Describe any methods used to examine subgroups and interactions | N/A |
| Statistical methods | #12c | Explain how missing data were addressed | N/A |
| Statistical methods | #12d | If applicable, describe analytical methods taking account of sampling strategy | N/A |
| Statistical methods | #12e | Describe any sensitivity analyses | N/A |
| **Results** | | | |
| Participants | #13a | Report numbers of individuals at each stage of study—eg numbers potentially eligible, examined for eligibility, confirmed eligible, included in the study, completing follow-up, and analysed. Give information separately for for exposed and unexposed groups if applicable. | 5 |
| Participants | #13b | Give reasons for non-participation at each stage | 5 |

For peer review only

| | | | |
|---|---|---|---|
| Participants | #13c | Consider use of a flow diagram | N/A |
| Descriptive data | #14a | Give characteristics of study participants (eg demographic, clinical, social) and information on exposures and potential confounders. Give information separately for exposed and unexposed groups if applicable. | Table 1, 3 |
| Descriptive data | #14b | Indicate number of participants with missing data for each variable of interest | N/A |
| Outcome data | #15 | Report numbers of outcome events or summary measures. Give information separately for exposed and unexposed groups if applicable. | N/A |
| Main results | #16a | Give unadjusted estimates and, if applicable, confounder-adjusted estimates and their precision (eg, 95% confidence interval). Make clear which confounders were adjusted for and why they were included | N/A |
| Main results | #16b | Report category boundaries when continuous variables were categorized | N/A |
| Main results | #16c | If relevant, consider translating estimates of relative risk into absolute risk for a meaningful time period | N/A |
| Other analyses | #17 | Report other analyses done—e.g., analyses of subgroups and interactions, and sensitivity analyses | N/A |

**Discussion**

| | | | |
|---|---|---|---|
| Key results | #18 | Summarise key results with reference to study objectives | 12 |

For peer review only

| | | | |
|---|---|---|---|
| Limitations | #19 | Discuss limitations of the study, taking into account sources of potential bias or imprecision. Discuss both direction and magnitude of any potential bias. | 12 |
| | #20 | Give a cautious overall interpretation considering objectives, limitations, multiplicity of analyses, results from similar studies, and other relevant evidence. | 12 |
| Generalisability | #21 | Discuss the generalisability (external validity) of the study results | 13 |
| **Other Information** | | | |
| Funding | #22 | Give the source of funding and the role of the funders for the present study and, if applicable, for the original study on which the present article is based | 1 |

None The STROBE checklist is distributed under the terms of the Creative Commons Attribution License CC-BY. This checklist can be completed online using https://www.goodreports.org/, a tool made by the EQUATOR Network in collaboration with Penelope.ai

For peer review only

For peer review only

## Standards for Reporting Qualitative Research (SRQR)*

http://www.equator-network.org/reporting-guidelines/srqr/

**Title and abstract**

| | Page/line no(s). |
|---|---|
| **Title** - Concise description of the nature and topic of the study Identifying the study as qualitative or indicating the approach (e.g., ethnography, grounded theory) or data collection methods (e.g., interview, focus group) is recommended | N/A |
| **Abstract** - Summary of key elements of the study using the abstract format of the intended publication; typically includes background, purpose, methods, results, and conclusions | 2 |

**Introduction**

| | |
|---|---|
| **Problem formulation** - Description and significance of the problem/phenomenon studied; review of relevant theory and empirical work; problem statement | 3 |
| **Purpose or research question** - Purpose of the study and specific objectives or questions | 3 |

**Methods**

| | |
|---|---|
| **Qualitative approach and research paradigm** - Qualitative approach (e.g., ethnography, grounded theory, case study, phenomenology, narrative research) and guiding theory if appropriate; identifying the research paradigm (e.g., postpositivist, constructivist/ interpretivist) is also recommended; rationale** | 4 |
| **Researcher characteristics and reflexivity** - Researchers' characteristics that may influence the research, including personal attributes, qualifications/experience, relationship with participants, assumptions, and/or presuppositions; potential or actual interaction between researchers' characteristics and the research questions, approach, methods, results, and/or transferability | No |
| **Context** - Setting/site and salient contextual factors; rationale** | 3 |
| **Sampling strategy** - How and why research participants, documents, or events were selected; criteria for deciding when no further sampling was necessary (e.g., sampling saturation); rationale** | 3/4 |
| **Ethical issues pertaining to human subjects** - Documentation of approval by an appropriate ethics review board and participant consent, or explanation for lack thereof; other confidentiality and data security issues | 3 |
| **Data collection methods** - Types of data collected; details of data collection procedures including (as appropriate) start and stop dates of data collection and analysis, iterative process, triangulation of sources/methods, and modification of procedures in response to evolving study findings; rationale** | 3,4 |
| **Data collection instruments and technologies** - Description of instruments (e.g., interview guides, questionnaires) and devices (e.g., audio recorders) used for data collection; if/how the instrument(s) changed over the course of the study | 4 |
| **Units of study** - Number and relevant characteristics of participants, documents, or events included in the study; level of participation (could be reported in results) | 4 |
| **Data processing** - Methods for processing data prior to and during analysis, including transcription, data entry, data management and security, | 4 |

For peer review only

| | |
|---|---|
| verification of data integrity, data coding, and anonymization/de-identification of excerpts | |
| **Data analysis** - Process by which inferences, themes, etc., were identified and developed, including the researchers involved in data analysis; usually references a specific paradigm or approach; rationale** | 4 |
| **Techniques to enhance trustworthiness** - Techniques to enhance trustworthiness and credibility of data analysis (e.g., member checking, audit trail, triangulation); rationale** | |

**Results/findings**

| | |
|---|---|
| **Synthesis and interpretation** - Main findings (e.g., interpretations, inferences, and themes); might include development of a theory or model, or integration with prior research or theory | 6 onwards |
| **Links to empirical data** - Evidence (e.g., quotes, field notes, text excerpts, photographs) to substantiate analytic findings | No |

**Discussion**

| | |
|---|---|
| **Integration with prior work, implications, transferability, and contribution(s) to the field -** Short summary of main findings; explanation of how findings and conclusions connect to, support, elaborate on, or challenge conclusions of earlier scholarship; discussion of scope of application/generalizability; identification of unique contribution(s) to scholarship in a discipline or field | 12,13 |
| **Limitations** - Trustworthiness and limitations of findings | 12 |

**Other**

| | |
|---|---|
| **Conflicts of interest** - Potential sources of influence or perceived influence on study conduct and conclusions; how these were managed | 1 |
| **Funding** - Sources of funding and other support; role of funders in data collection, interpretation, and reporting | 1 |

*The authors created the SRQR by searching the literature to identify guidelines, reporting standards, and critical appraisal criteria for qualitative research; reviewing the reference lists of retrieved sources; and contacting experts to gain feedback. The SRQR aims to improve the transparency of all aspects of qualitative research by providing clear standards for reporting qualitative research.

For peer review only

**The rationale should briefly discuss the justification for choosing that theory, approach, method, or technique rather than other options available, the assumptions and limitations implicit in those choices, and how those choices influence study conclusions and transferability. As appropriate, the rationale for several items might be discussed together.

**Reference:**

O'Brien BC, Harris IB, Beckman TJ, Reed DA, Cook DA. **Standards for reporting qualitative research: a synthesis of recommendations.** *Academic Medicine*, Vol. 89, No. 9 / Sept 2014

DOI: 10.1097/ACM.0000000000000388

For peer review only
